# An integral genomic signature approach for tailored cancer therapy using genome-wide sequencing data

**DOI:** 10.1038/s41467-022-30449-7

**Published:** 2022-05-26

**Authors:** Xiao-Song Wang, Sanghoon Lee, Han Zhang, Gong Tang, Yue Wang

**Affiliations:** 1 UPMC Hillman Cancer Center, University of Pittsburgh, Pittsburgh, PA 15213 USA; 2 Department of Pathology, University of Pittsburgh, Pittsburgh, PA 15213 USA; 3 Department of Biomedical Informatics, University of Pittsburgh, Pittsburgh, PA 15206 USA; 4 Department of Biostatistics, University of Pittsburgh, Pittsburgh, PA 15261 USA

**Keywords:** Cancer models, Targeted therapies, Genome informatics, Cancer genomics, Statistical methods

## Abstract

Low-cost multi-omics sequencing is expected to become clinical routine and transform precision oncology. Viable computational methods that can facilitate tailored intervention while tolerating sequencing biases are in high demand. Here we propose a class of transparent and interpretable computational methods called integral genomic signature (iGenSig) analyses, that address the challenges of cross-dataset modeling through leveraging information redundancies within high-dimensional genomic features, averaging feature weights to prevent overweighing, and extracting unbiased genomic information from large tumor cohorts. Using genomic dataset of chemical perturbations, we develop a battery of iGenSig models for predicting cancer drug responses, and validate the models using independent cell-line and clinical datasets. The iGenSig models for five drugs demonstrate predictive values in six clinical studies, among which the Erlotinib and 5-FU models significantly predict therapeutic responses in three studies, offering clinically relevant insights into their inverse predictive signature pathways. Together, iGenSig provides a computational framework to facilitate tailored cancer therapy based on multi-omics data.

## Abstract (web summary)

Predicting drug responses in cancer patients requires robust computational frameworks. Here, the authors develop an integral genomic signature —iGenSig— approach to predict drug responses using multi-omics data from tumour samples, and validate this approach using genomic datasets from multiple clinical studies.

## Introduction

Precision oncology, defined as molecular profiling of tumors to achieve customized patient care, has entered the mainstream of cancer patient care^1^. The current standard practices for precision oncology include detecting actionable mutations via genetic testing (i.e., *EGFR* mutation, *ALK* rearrangements), or detecting small-sized predictive or prognostic gene signatures via targeted expression assays (i.e., Oncotype DX, MammaPrint). Such assays, however, require at least one assay per decision, which limit their cost-effectiveness. On the other hand, the past ten years have observed a stunning reduction of sequencing costs for a human genome from $300,000 to $1000, with $100 whole-genome sequencing expected soon^2^. With this rate, it is expected that transcriptome and genome sequencing will become the clinical routine for patients. With the advent of low-cost genome sequencing, precision oncology is at the cusp of a deep transformation via leveraging the big data to provide a wide array of clinical decision supports which is deemed to be cost-effective. The computational approaches that can leverage these big data to facilitate clinical decisions and provide tailored health care are in high demand. For example, in metastatic lung cancer, the target therapies prescribed based on the current modeling of genomic sequencing data produced only minimal gain of quality-adjusted life year^3^. Innovative and robust clinical big data-based decision support models for precision oncology will be of vital importance.

In recent years, there has been great enthusiasm about the potential of artificial intelligence-based clinical decision support systems for big data-based precision medicine, however, to date only few examples exist that impact clinical practice^4^. The main challenge is that multi-OMIC big data typically contain daunting amounts of high-dimensional features but a limited number of subjects which poses great challenges to the computational power and training process of artificial intelligence (AI) -based methods. In addition, AI approaches are “black box” tools, so the algorithmic and biological mechanisms underlying the models are largely unknown. The modeling process is controlled by AI which makes it difficult to interpret complex model predictions and is often plagued with the problems of overfitting and overweighing. In addition, there is a lack of big data-based methods specifically addressing the insufficient performance of the prediction models for crossing dataset modeling resulting from the common biases in detected genomic features across different datasets arising from sequencing errors, different library preparation methods and platforms, discordant sequencing depth and read-length, heterogeneous sample qualities, and experimental variations, etc. This calls for robust, transparent, and explainable methods that can predict clinical treatment outcomes from multi-OMIC data with substantially improved tolerance of sequencing biases.

**Fig. 1:**
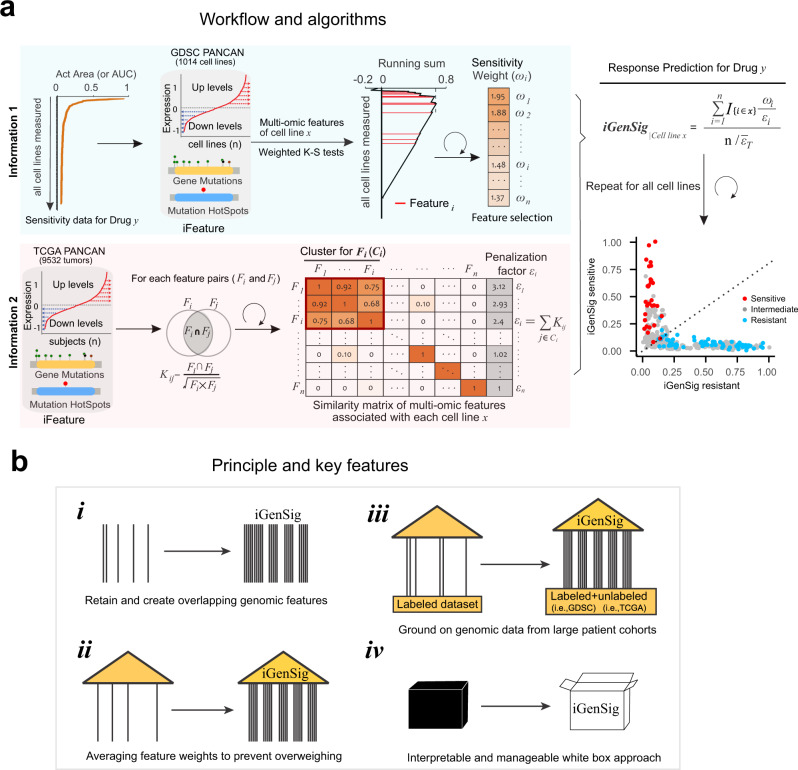
The principle, workflow, and algorithms of the integral genomic signature modeling approach. **a** The workflow and algorithms of integral genomic signature analysis. The upper panel shows the calculation of the weights for significant genomic features that predict drug sensitivity or resistance based on weighted K-S tests of Act Area or AUC for each drug respectively, and the lower panel shows the computation of a similarity matrix for genomic features based on TCGA Pan-Cancer dataset to penalize the redundancy between the genomic features associated with each cell line *x*. The resulting sensitive or resistant genomic signature scores are calculated separately using the weights predictive of sensitivity or resistance respectively based on the indicated formula. The dot plot shows the sensitive and resistant iGenSig scores for all cell line subjects, with red and blue colors indicating sensitive and resistant cell lines. **b** Schematic showing the principle and key features of iGenSig modeling: (i) the iGenSig approach intentionally retains and creates redundant genomic features, a concept like the use of redundant steel rods to reinforce the pillars of a building. (ii) iGenSig modeling utilizes the average correlation intensities of significant genomic features detected in specific samples to diminish the effect of false-positive detection resulting from sequencing errors and prevent overweighing. (iii) iGenSig modeling extracts the second genomic information from unlabeled genomic datasets for large cohorts of human cancers, in addition to the labeled genomic datasets of drug sensitivity, which will substantially improve its cross-dataset applicability, particularly on clinical trial datasets. (iv) iGenSig modeling is a white-box approach, thus will be more interpretable and controllable than machine learning or deep learning approaches.

In this work, we propose a class of methods for big data-based precision medicine called integral genomic signature (iGenSig) analysis, which is designed to provide more robust clinical decision support with higher transparency and cross-dataset applicability (Fig. 1). Due to the computational challenge of the high dimensionality of genomic features, a common practice for big data-based modeling is to reduce the dimensionality of genomic features via removing redundant variables highly correlative with each other for gene expression signature panels, or creating synthetic features for machine learning approaches^5^ (Supplementary Fig. 1). Here we propose that the redundancies within high-dimensional features can in fact overcome sequencing errors and bias especially when there is a loss of detection of a subset of correlates. Here we define the genomic features significantly predicting a clinical phenotype (such as therapeutic response) as genomic correlates, and an integral genomic signature as the integral set of redundant high-dimensional genomic correlates for a given clinical phenotype such as therapeutic response. The iGenSig analysis generates prediction scores based on the set of redundant genomic features from labeled genomic datasets of therapeutic responses, and then reduces the effect of feature redundancy via adaptively penalizing the redundant features detected in specific samples based on their co-occurrence assessed using unlabeled genomic datasets for large cohorts of human cancers from The Cancer Genome Atlas (TCGA) (Fig. 1a). This allows for preserving redundant genomic features and introducing de novo redundant genomic features during the modeling while preventing the feature redundancy from flattening the scoring system. With this method, we speculate that if a subset of the genomic features was lost due to sequencing biases or experimental variations, the redundant genomic features would help sustain the prediction score. More importantly, we expect that the unbiased genomic information obtained from unlabeled large cancer cohorts will substantially improve the cross-dataset applicability of the iGenSig models, particularly on clinical trial datasets. On the other hand, iGenSig modeling utilizes the average correlation intensities of significant genomic features detected in specific samples to diminish the effect of false-positive detection resulting from sequencing errors and overweighing. This method also prevents overfitting by dynamically adjusting the feature weights for training subjects. Thus, iGenSig is a simple, white-box solution with an integral design to tolerate sequencing errors and bias for big data-based precision medicine. The principle and key features of iGenSig modeling are summarized in Fig. 1b.

## Results

### Development of the integral genomic signature approach based on a genomic dataset of drug sensitivity

To develop the iGenSig modeling, we utilized the drug sensitivity measurements of chemical perturbations, gene expression profiling data, and exome sequencing data for 989 cancer cell lines released by Genomic Datasets of Drug Sensitivity^6^ (GDSC, Supplementary Table 1). For the drug response measurements, we used high Act Area, the area above the fitted dose-response curve (or 1-AUC), to define a sensitive drug response, and high AUC, the area under the dose curve, to define a resistant response. According to the literature, the AUC and Act Area are much better quantifiers of drug responses than IC50^7^. To uniform multi-OMIC features, we formulated a Genomic-feature Matrix Transposed (GMT) format for compiling binary multi-OMIC features, similar to that used for compiling gene concepts^8,9^. Using this format, we analyzed the expression profiling data and exome sequencing data from GDSC and compiled an integrated dataset combining the genomic features including upregulated genes, downregulated genes, mutated genes, and mutation hotspots. To increase the cross-dataset applicability of the iGenSig models, we intentionally introduced de novo feature redundancy by generating twelve overlapping levels of differentially expressed gene lists (Fig. 1a). We then selected significant genomic correlates using a weighted Kolmogorov–Smirnov (K-S) test that ranks the enrichment of each genomic feature in the cell line panel sorted decreasingly by Act Area or AUC, similar to that implemented by Gene Set Enrichment Analysis (GSEA)^10^. Next, we leveraged the TCGA Pan-Cancer RNAseq and exome dataset for 9532 tumors to quantify the co-occurrence between genomic features associated with each cell line based on similarity measures, which were then used to calculate a redundancy penalty score for each genomic feature. This provides unbiased information about the feature redundancy based on unlabeled extra-large patient cohorts.

**Fig. 2:**
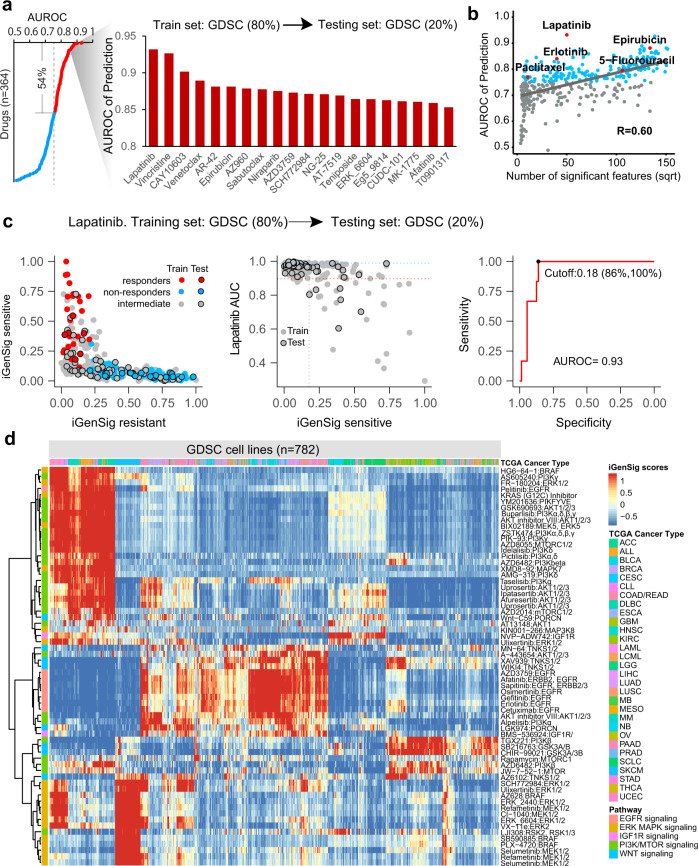
The performance of iGenSig models in predicting the drug responses of GDSC cell lines. **a** The performance of the iGenSig models for GDSC profiled drugs was assessed by their average AUROC. About 364 drugs that show a negatively skewed drug response distribution in cancer cell lines and have at least 20 sensitive cell lines are included in the analyses. The drugs with top-performing models (AUROC >0.85) are shown in bar chart on the right. The average AUROC for each drug was calculated based on five train/test sets. **b** Correlating the performance of the iGenSig models for 364 drugs with their average number of significant genomic features. The drug models assessed on the six clinical trial datasets are highlighted in red. **c** the performance of the iGenSig model for Lapatinib in predicting the response of GDSC cell lines based on a representative training and testing set. Left, sensitive, and resistant GenSig scores for GDSC cell lines. Middle, the correlation of the iGenSig scores with AUC measurements for Lapatinib. Right, the receiver operating characteristic (ROC) curve for predicting sensitive responses to Lapatinib. As the golden standard for the ROC curve, the cell line subjects in the test set are divided into sensitive and non-sensitive groups based on the AUC measurements for Lapatinib using the cutoff determined by the waterfall method (see Methods). **d** Ward D2 Hierarchical Clustering for GDSC cancer cell lines and targeted kinase drugs of five RTK signalings based on iGenSig scores. The drugs targeting different kinases or different kinase families form distinctive clusters.

To prevent the bias from overfitting, we used a random collection of 80% GDSC cell lines as a train set and the rest 20% as an internal test set for assessing the performance of the model. A total of five train/test sets are generated for modeling through random permutations. We then performed iGenSig modeling for 364 drugs that elicit a negatively skewed drug response distribution in cancer cell lines indicating the narrow effect of outstanding responses and have at least 20 sensitive cell line subjects indicating the availability of outstanding responders. To benchmark the performance of the models, we discretized the cell lines into drug-sensitive and non-sensitive groups based on a waterfall method established in a previous study^11^, and calculated the Area Under ROC Curve (AUROC) for each drug. As a result, 196 drugs showed an AUROC > 0.75 on the testing sets (54%), and 20 drugs showed a ROCAUC >0.85 (Fig. 2a and Supplementary Data 1). Many of the top-performing drugs are FDA-approved chemotherapy or targeted therapy agents for cancer treatment, such as Lapatinib, Vincristine, Venetoclax, Epirubicin, Niraparib, and Afatinib. The top-performing drug models include targeted therapies against well-known cancer targets such as ERBBs, HDAC, BCL2, JAKs, PARP, ERK, CDKs, etc., and Lapatinib, Vincristine, and CAY10603 presented the best performing models with an average AUROC more than 0.9. The predictive powers of the iGenSig models appear to obviously correlate with the number of available genomic correlates for each drug (Spearman *R* = 0.60, Fig. 2b), suggesting that the iGenSig models rely on the available genomic information that can predict drug responses. The iGenSig scores negatively correlate with the AUC drug measurements in cell lines with a similar trend in both training and testing sets as exemplified by the Lapatinib model (Fig. 2c), suggesting that iGenSig modeling do not overfit toward the training set. Next, we clustered the drug's target kinase signaling based on their iGenSig scores in GDSC cell lines, which resulted in distinctive clustering of the drugs targeting the same or similar kinases (Fig. 2d). Interestingly, the Pan-cancer cell lines form five distinctive sensitivity clusters toward the drugs targeting the five kinase pathways. Outstanding response predictions for BRAF/MEK inhibitors are preferentially enriched in melanoma cell lines, while other drugs such as EGFR inhibitors exhibit cancer-type agnostic iGenSig scores, consistent with the tumor-type related clinical activities of these drugs.

### iGenSig models show no performance loss on the independent validation dataset for drug sensitivity

**Fig. 3:**
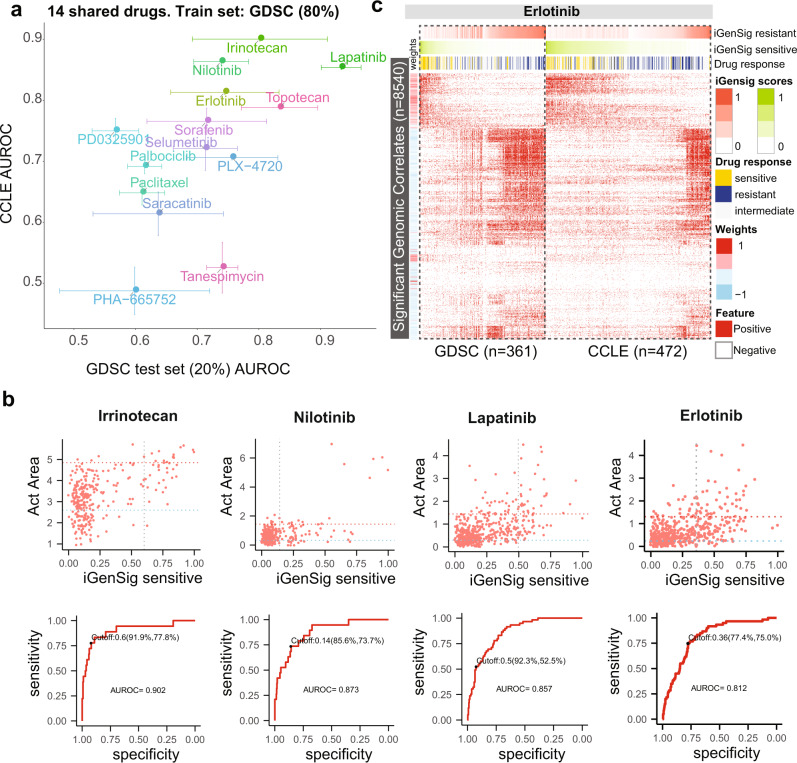
Predictive values of iGenSig models developed from the GDSC pharmacogenomic dataset on the sensitivity of CCLE profiled cell lines to the shared drugs. **a** The performance of GDSC iGenSig models in predicting the responses of GDSC testing cell lines and CCLE cell lines to 14 drugs shared between the two datasets. 80% of GDSC cell lines are used for building the iGenSig models and 20% of GDSC cell lines are used for testing. 100% of CCLE cell lines are used for cross-dataset validations. If the same drug is profiled by both GDSC batch 1 and 2, the drug sensitivity data from batch 1 are used in the analysis. The error bars show standard deviations. **b** The predictive values of the iGenSig models developed from GDSC data on the CCLE cell lines treated with Irinotecan, Nilotinib, Lapatinib, or Erlotinib. The upper panel shows the correlation between the iGenSig scores and the Act Areas of the respective drugs for CCLE cell lines. The horizontal dashed lines show the cut-offs for sensitive (red) and resistant (blue) calls. The vertical dashed line shows the optimal cut-off for iGenSig scores determined based on AUROC. The lower panel shows the ROC curves of iGenSig scores in determining the sensitive cell lines vs non-sensitive cell lines. **c** GDSC and CCLE cell lines show consistent integral genomic signature that correlates with Erlotinib responses. The significant genomic features (*n* = 8540) based on K-S tests are shown in the figure. The GDSC and CCLE cell lines are first sorted by their sensitive iGenSig scores; the cell lines with sensitive iGenSig scores less than the median are then sorted by the resistant iGenSig cores. The cell lines that have been tested for Erlotinib chemical perturbations are shown in the figure, and the sensitive and resistant cell line subjects are indicated as yellow and blue bars.

To assess the cross-dataset performance of our iGenSig models, we analyzed the RNAseq and exome sequencing data from the Cancer Cell Line Encyclopedia (CCLE)^11^. In total there are 14 drugs measured by both CCLE and GDSC datasets. Our result showed that the predictive performance of iGenSig models on the CCLE dataset appears to correlate with their performance on the testing sets of the GDSC dataset (Pearson *R* = 0.58, Fig. 3a). Using GDSC as a training set and CCLE as a validation set, the models for four drugs achieved AUROC of more than 0.8. These include Irinotecan, Nilotinib, Lapatinib, and Erlotinib, for which the AUROC for prediction are 0.902, 0.873, 0.857, and 0.812 respectively (Fig. 3b). Plotting the significant genomic features for Erlotinib in the two datasets revealed a consistent integral genomic signature correlating with drug-sensitive or resistant responses (Fig. 3c), as opposed to the modest consistency of the drug sensitivity measurements^12^. This is in line with the previous report about the greater agreement of prediction models than raw sensitivity values^13^, which could be attributed to the number of cell lines screened by both GDSC and CCLE for which insufficient sensitive cell lines were screened in both projects^14^, or due to the difference in cellular states under different cell culture conditions. It is interesting to note that the predictive performance of iGenSig models resulting from the permutated training sets on the CCLE validation dataset showed much lower deviations compared to that on the GDSC testing dataset (Fig. 3a). This may be attributed to the much smaller number of sensitive subjects in the GDSC testing datasets compared to the CCLE validation dataset.

To test if the iGenSig predictions rely on the genomic features of the primary drug targets, we removed the drug target genes for Erlotinib, Lapatinib, or Nilotinib from GDSC and CCLE genomic feature sets. We then built the iGenSig models for these drugs based on the genomic features devoid of drug targets and assessed their performance on GDSC internal test set or the CCLE validation set. Our result showed that the performances of these iGenSig models are not affected by deleting the genomic features for known drug targets (Supplementary Fig. 2a). Furthermore, we examined if excluding the hematologic cancer cell lines such as leukemia and lymphoma from the GDSC training dataset can improve the prediction performances of iGenSig models on the drug sensitivity of CCLE solid cancer cell lines. Our results, however, did not significantly improve the performance of the fourteen drug models, but instead, this approach slightly decreased the overall performance (Supplementary Fig. 2b). This suggests that there may be predictive genomic information gained from these hematologic cancer cell lines as well. We thus used the models developed from the Pan-cancer cell line dataset in the following analysis.

### The iGenSig model predicts the response of patient subjects to Erlotinib treatment in the BATTLE trial and SAKK 19/05 trial

**Fig. 4:**
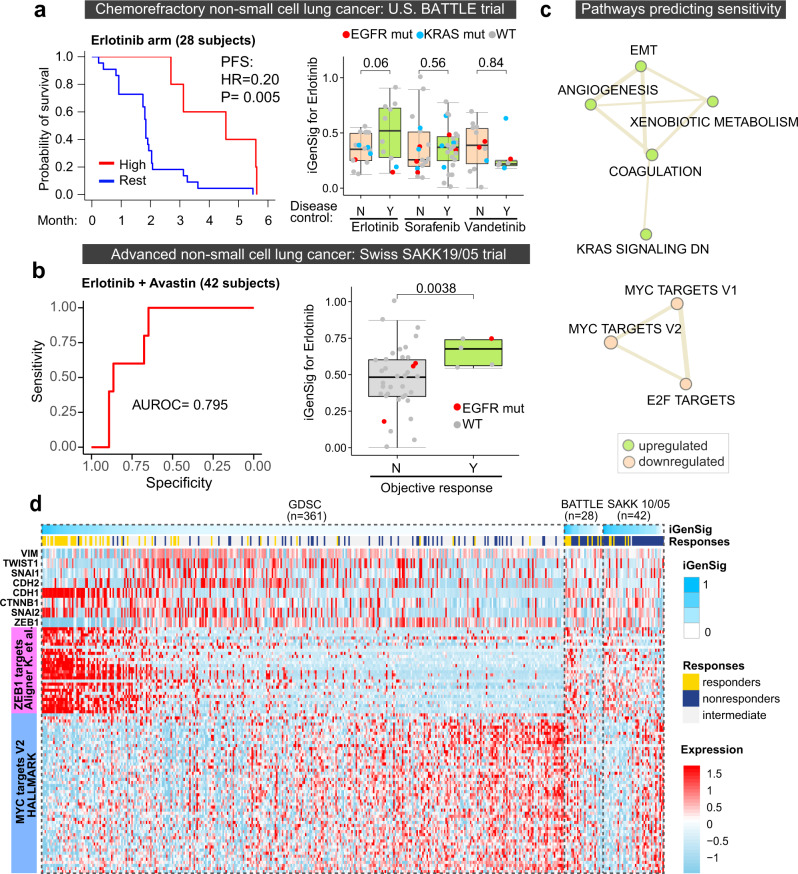
Predictive values of the iGenSig model for Erlotinib developed from GDSC cell line pharmacogenomic data on the survival of patient subjects from the US BATTLE trial and Swiss SAKK 19/05 trial. **a** Left, Kaplan–Meier plot showing the predictive values of GDSC iGenSig model for Erlotinib on the patients from the U.S. BATTLE trial. A data-driven cut point of high iGenSig scores was determined as described in Methods. The *P* value is based on the log-rank test. Right, the differences in iGenSig scores among patients that achieved (Y) or did not achieve (N) 8-week disease control in the Erlotinib, Sorafenib, and Vandetanib treatment arms. Patients with EGFR or KRAS mutations are depicted with red or blue colors. **b** The predictive values of the GDSC iGenSig model for Erlotinib on the patient subjects from the Swiss SAKK 19/05 trial. Left, the ROC curve showing the performance of sensitive iGenSig scores on predicting the objective responses of patient subjects at 12 weeks following Erlotinib and Avastin treatment in the Swiss SAKK 19/05 clinical trial. Right, the predictive value of the iGenSig model for Erlotinib does not depend on EGFR mutation status. The box plots in **a**, **b** show the minima, first quartile, median, third quartile, and the maxima. **c** The network of upregulated and downregulated pathways characteristic of Erlotinib sensitive GDSC cell line subjects. The top upregulated and downregulated pathways clustered in the respective interconnected networks are shown in the figure. The CSEA enrichment score for each pathway in the Erlotinib sensitive signature is depicted by the size of each node. The pathway associations are depicted by the thickness of the edge. The pathway associations are calculated based on CSEA association scores between each pair of pathways. **d** Heatmap showing the associations of EMT markers and master transcription factors, as well as ZEB1 and MYC target genes with the sensitive iGenSig scores for Erlotinib in the GDSC, BATTLE, and SAKK 10/05 clinical trial datasets. The cell lines and patient subjects are sorted based on their sensitive iGenSig scores. The boxplot elements in 4a and 4b indicate the max, 75th percentile, median, 25th percentile, and min. The *p* values shown in the box plots of 4a, b are based on one-sided student’s *t*-tests.

Next, we sought to test the applicability of the GDSC iGenSig models in predicting therapeutic responses of patient subjects in clinical trials. We first focused on EGFR inhibitors for which our iGenSig models showed excellent cross-dataset performance. Most of the clinical trials for targeted drugs assessed their combinations with chemotherapies instead of monotherapies, which we postulate could confound the outcome of drug response prediction. Our further literature investigation revealed that a genomic study of the BATTLE trial (GSE33072) profiled non-small cell lung cancer (NSCLC) tumors from 131 patients by gene expression array, among which 28 patients are treated with Erlotinib monotherapy, 47 patients are treated with Sorafenib monotherapy, and 20 patients are treated with vandetanib. Overall, the patient responses to Erlotinib in this trial are limited, and all patients treated with Erlotinib progressed within six months. This may be due to the selection of pretreated chemorefractory NSCLC patients as enrollment criteria^15^. Despite this, progression-free survival (PFS) analysis suggested that our GDSC iGenSig model for Erlotinib significantly predicted the favorable response of these patients in the Erlotinib arm, with a hazard ratio of 0.2 (*p* = 0.005, Fig. 4a, left). Among the three major treatment arms of this trial, the GDSC Erlotinib model showed a specific predictive effect on the Erlotinib arm compared to the Sorafenib or Vandetinib arms (Fig. 4a, right).

Next, we examined the predictive value of this model on a Swiss SAKK 19/05 trial that tested the combination of Erlotinib and bevacizumab (Avastin)^16^. Recent evidence suggested that the addition of Bevacizumab to Erlotinib exhibits increased therapeutic efficacy. As bevacizumab alone is known to lack efficacy in lung cancer, this effect is thought to be the result of enhanced erlotinib activity^16^. The SAKK 19/05 trial is a multi-center single-arm trial in previously untreated patients. The endpoint provided by this study is objective response at 12 weeks after Erlotinib and bevacizumab treatment, and no survival data are available. Our result showed that the GDSC iGenSig model for Erlotinib showed a predictive AUROC of 0.795 (Fig. 4b, left), and this predictive value is independent of *EGFR* mutation status (Fig. 4b, right). On the other hand, out of the four patients with *EGFR* mutated tumors, only the tumor showing the highest iGenSig score exhibited an objective response. This suggests that while EGFR inhibition is indicated for *EGFR* mutated patients, a subgroup of *EGFR* wild-type patients may derive significant benefit from EGFR inhibitors as well, which could be identifiable by the iGenSig model. Consistently, clinical studies suggest that EGFR inhibition should not be confined to *EGFR* mutated lung cancer patients^17^. In addition to clinical trial datasets, we also applied our iGenSig model to a set of PDX models treated with Erlotinib and profiled with RNAseq and WXS, which revealed significant predictive value as well (HR = 0.12, *p* = 0.0001, Supplementary Fig. 3). Taken together, these results support the utility of integral genomic signature modeling in predicting therapeutic responses of EGFR inhibition and its excellent cross-dataset performance.

### Biological interpretation of the iGenSig model yielded insights into the predictive signature pathways

Since epithelial-mesenchymal transition (EMT) has been previously reported to mediate EGFR resistance in the BATTLE trial study^18^, we wonder if the EMT signature contributes to the iGenSig predictions. We thus examined the pathways characteristic of the integral genomic signature for Erlotinib sensitivity in our iGenSig model. This can be achieved by extracting the genes contributing to the genomic features predicting sensitive responses in our GDSC iGenSig model. The resulting gene list can be then used to explore the enriched pathways based on the concept signature enrichment analysis (CSEA) developed in our previous study, which is designed for deep functional assessment of the pathways enriched in an experimental gene list^9^. Our result showed that the most significantly downregulated pathways characteristic of Erlotinib sensitive responses include MYC and E2F target gene signatures (Fig. 4c). Consistent with this, amplification of *MYC* has been found to mediate resistance to EGFR inhibitors, and targeting MYC was proposed as a promising strategy to overcome acquired resistance^19^. On the other hand, the EMT pathway is ranked as one of the most significantly upregulated pathways in the Erlotinib sensitive signature identified from GDSC cell lines, which contradicts the previous report^18^.

We postulated that this may be attributed to the content of the EMT signature that mixed both upregulated and downregulated genes in EMT. We thus compiled an upregulated EMT signature and a downregulated EMT signature based on a previous report^20^. Correlating these EMT signatures with the Erlotinib iGenSig scores revealed that the downregulated and upregulated EMT signatures are indeed enriched in the subjects with high or low iGenSig scores respectively in the BATTLE trial dataset (Supplementary Fig. 4). However, in the GDSC Pan-Cancer cell line panel, both upregulated and downregulated EMT signatures are repressed in Erlotinib-resistant cell lines. This suggests that the repressions of both EMT signatures are characteristic of the Erlotinib-resistant cell lines at the Pan-cancer scale and explains the contradicting pathway enrichment results from CSEA analysis. Among the known EMT markers and transcription factors, overexpression of E-cadherin (CDH1) was observed in both sensitive cell lines and patient responders from BATTLE trial (Fig. 4d). Whereas overexpression of EMT markers such as N-cadherin (CDH2), Vimentin (VIM), and β-catenin (CTNNB1) are characteristic of the cell lines with intermediate sensitivity, and overexpression of ZEB1 is characteristic of Erlotinib-resistant cell lines. In the BATTLE trial, overexpression of β-catenin and/or ZEB1 are characteristic of subjects with low iGenSig scores. As ZEB1 is a transcriptional repressor, we assessed the correlation of ZEB1 target genes with the iGenSig scores. This revealed that the downregulation of ZEB1 target genes is characteristic of both resistant cell lines and patient subjects in the two clinical trials (Fig. 4d). On the other hand, MYC target genes appear to be upregulated in the most Erlotinib-resistant cell lines. Together, our results suggest that EMT is associated with reduced but intermediate response to Erlotinib whereas repression of ZEB1 signature and upregulation of MYC signature is associated with tumor-type agnostic resistance at the Pan-cancer scale. It is interesting to note that the iGenSig scores for the BATTLE trial appear to accentuate the epithelial signature on high-scored subjects, whereas the iGenSig scores for the SAKK 10/05 trial accentuate the MYC signature on low-scored subjects. This may explain their better performance in predicting sensitive or resistant subjects respectively (see Discussion).

### The iGenSig model predicted patient response to 5-FU treatment in a French CIT multi-center study

**Fig. 5:**
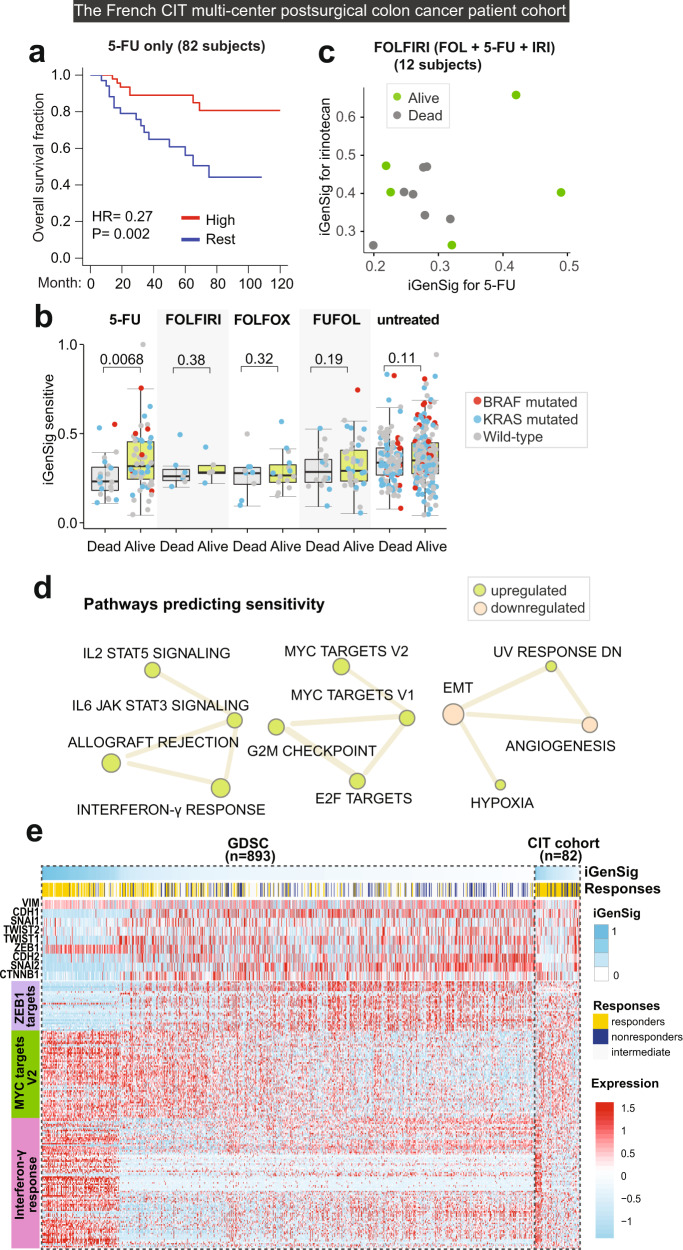
Predictive values of the iGenSig model for 5-FU developed from GDSC cell line pharmacogenomic dataset on patient survival in the French CIT multicenter postsurgical colon cancer patient cohort. **a** Kaplan–Meier plot showing the predictive values of the GDSC iGenSig model for 5-FU on the overall survival of patients from the French CIT cohort treated with 5-FU monotherapy. A data-driven cut point of high iGenSig scores was determined as described in Methods and the *P* value is calculated based on the log-rank test. **b** The predictive values of the GDSC iGenSig model for 5-FU on the overall survival of all patient subjects from the CIT cohort treated with different adjuvant chemotherapy regimens or untreated. The BRAF and KRAS mutation status for each subject are indicated by colored dots. The boxplot elements indicate the maxima, 75th percentile, median, 25th percentile, and minima. The *p* values are based on one-sided student’s *t*-tests. **c** The predictive values of the GDSC iGenSig models for irinotecan and 5-FU on the patient subjects treated with the FOLFIRI regimen in the CIT study. The patients are stratified based on their overall survival and their sensitive iGenSig scores based on the two models are plotted. **d** The network of upregulated and downregulated pathways characteristic of 5-FU sensitive GDSC cell line subjects. The top upregulated and downregulated pathways clustered in the respective interconnected networks are shown in the figure. The CSEA enrichment score for each pathway in the Erlotinib sensitive signature is depicted by the size of each node. The pathway associations are depicted by the thickness of the edge. The pathway associations are calculated based on CSEA association scores between each pair of pathways. **e** Heatmap showing the associations of EMT markers and master transcription factors, ZEB1 and MYC target genes, and interferon γ responsive genes with the sensitive iGenSig scores for 5-FU in the GDSC and CIT datasets. The cell lines and patient subjects are sorted based on their sensitive iGenSig scores for 5-FU.

Next, we sought to examine the utility of iGenSig modeling in predicting chemotherapy response. While most clinical studies of chemo-agents focus on testing combination regimens, we identified a multicenter clinical study carried out by the French Cartes d’Identité des Tumeurs (CIT) program that tested 5- Fluorouracil (5-FU) monotherapy on postsurgical colon cancer patient^21^. In addition, this study also tested combination chemotherapy regimens such as FOLFIRI, FOLFOX, and FUFOL. 5-FU is an antimetabolite drug, and is one of the most commonly used drugs for cancer treatment, particularly for colorectal cancer^22^. The GDSC iGenSig model for 5-FU significantly predicted patient overall survival in the 5-FU monotherapy arm (*p* = 0.002), with a hazard ratio of 0.27 (Fig. 5a). Whereas this predictive effect was diminished in the treatment arms testing combination chemotherapies containing 5-FU (Fig. 5b). To examine if this is due to the therapeutic effect exerted by other chemo-agents, we examined the FOLFIRI arm testing the combination of folinic acid, 5-FU, and irinotecan, for which the iGenSig models for the latter two drugs are available. Among the alive patients in this arm, two patients showed high iGenSig scores by both models, whereas the other three patients showed high scores by either of these two models (Fig. 5c). This suggests that in the two alive patients with low 5-FU iGenSig scores, the therapeutic effects may be derived from irinotecan.

Next, we examined the pathways enriched in the 5-FU sensitive iGenSig signature. Interestingly, as opposed to the Erlotinib signature, the EMT pathway is ranked as the most downregulated pathway in the sensitive GDSC cell lines, whereas the MYC target gene signature and interferon γ signature are revealed as top upregulated pathways associated with sensitive responses (Fig. 5d, e). This suggests that the tumors that are resistant to EGFR inhibitors may be sensitive to the 5-FU treatment. Fascinatingly, consistent with this, it is known that EGFR wild-type tumors show higher sensitivity to uracil-tegafur than *EGFR* mutated tumors in lung cancer^23^, whereas EGFR inhibition has been found to sensitize 5-Fu-resistant colon cancer cells^24^. Moreover, the interferon γ signature is associated with an inflammatory response triggered by the double-strand breaks resulting from the DNA damaging effect of 5-FU. The upregulation of Interferon γ regulated genes in cancer cells may confer better therapeutic effects through the interferon γ induced growth arrest and apoptosis in cancer cells^25,26^, and this signature appears to be captured from leukemia and lymphoma cell lines in the GDSC panel (Supplementary Fig. 5).

### Clinical studies of combination drug therapies revealed confounding factors of iGenSig models

**Fig. 6:**
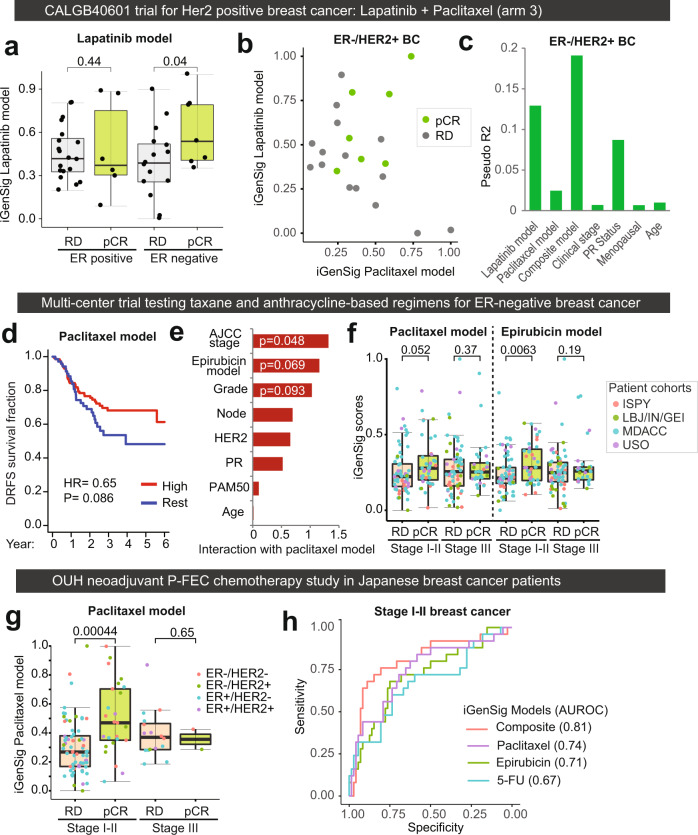
The predictive values of the GDSC iGenSig models on the survival of patient subjects from the CALGB40601 trial treated with lapatinib and paclitaxel, and from neoadjuvant clinical studies testing taxane-anthracycline-based combination chemotherapies. **a** The predictive values of the GDSC iGenSig model for lapatinib on the pathological responses (breast and axilla) of the patient subjects from arm 3 (lapatinib + paclitaxel) of the CALGB40601 trial. The patients are stratified based on their ER positivity. **b** The associations of the GDSC iGenSig models for lapatinib and paclitaxel with the pathological responses (breast and axilla) of the ER-negative patient subjects from arm 3 of the CALGB40601 trial. **c** Pseudo R2 showing the percentage of contribution explaining the variance in pathological responses of ER-negative patient subjects for GDSC lapatinib and paclitaxel models, their composite model, and other confounding clinical variables. **d** Kaplan–Meier plot showing the predictive values of the GDSC iGenSig model for Paclitaxel on the distant recurrence-free survival of patients with basal-like TNBC treated with neoadjuvant taxane-anthracycline chemotherapy. A data-driven cut point of high iGenSig scores was determined as described in Methods and the *P* value is determined based on the log-rank test. **e** The interactions of confounding clinical variables with the GDSC paclitaxel model were assessed based on multiple logistic regression models for overall survival status. The bar plot shows the −log10 transformed *p* values of Chi-Square tests comparing the pair-wise multiple logistic regression models with the simple logistic regression model for the GDSC paclitaxel model. **f** The predictive value of the GDSC iGenSig model for paclitaxel and Epirubicin on the pathological responses of the enrolled patient subjects stratified based on tumor stages. The source cohorts of patient subjects are indicated by colored dots. **g** The predictive values of the GDSC paclitaxel model on the pathological complete response of breast cancer patients to P-FEC chemotherapy stratified based on tumor stage. The receptor status of each patient is indicated by colored dots. **h** The AUROC of the GDSC models for Paclitaxel, Epirubicin, and 5-FU or their composite model on predicting the pathological complete responses of stage I-II breast cancer patients treated with P-FEC in the OUH study. The *p* values annotated on panels **a**, **f**, and **g** are based on one-sided student’s *t*-tests. The boxplot elements in 6a, 6f, and 6g indicate the maxima, 75th percentile, median, 25th percentile, and minima.

We then went on to further explore the predictive values of GDSC iGenSig models on the clinical trials testing the combination of targeted therapy with chemotherapy, or combinatory chemotherapy regimens. To achieve this, we identified three large gene expression datasets from breast cancer clinical studies testing the drug combinations containing the drugs with favorable GDSC iGenSig models. We first examined the predictive value of the lapatinib model on the CALGB40601 trial, a neoadjuvant phase III trial that tested the combination of Lapatinib with Paclitaxel in Her2-positive breast cancer in one of the three treatment arms^27^. As we expected, our results showed that the GDSC iGenSig models derived from lapatinib or paclitaxel single-drug treatment has limited predictive effects on these combination treatments, with an AUROC of 0.61 and 0.52 respectively. Since overexpression of estrogen receptor (ER) is known to confer resistance to HER2-targeted therapy through driving HER2-independent signaling^28^, thus may confound the predictive effect of our models, we stratified the patients based on their ER positivity. Following stratification, the lapatinib model showed significant predictive value in ER-negative patients (*p* = 0.04, Fig. 6a). In contrast, the paclitaxel model showed only a modest association with the pathological response (pCR) (Fig. 6b). This may be explained by the major clinical benefits derived from the HER2-targeted agents in HER2-positive patients. Next, we examined the predictive contributions of lapatinib and paclitaxel models as well as other clinical variables such as tumor stage, PR status, menopausal status, and patient age on patient outcome. By calculating Pseudo-R^2^ using logistic regression models (see Methods), the lapatinib model ranks the highest in explaining the variation in patient response, followed by PR status, and the composite model based on both lapatinib and paclitaxel models produced a higher predictive value than the individual models (Fig. 6c).

Next, we examined the predictive effect of the paclitaxel model on a prospective neoadjuvant multicenter clinical study for stage I-III ER-negative breast cancer patient cohort treated with chemotherapy containing sequential taxane and anthracycline-based regimens^29^. The GDSC paclitaxel model showed a moderate predictive effect on distant recurrence-free survival (*p* = 0.085), with a hazard ratio of 0.65 (Fig. 6d). Next, we sought to examine if anthracycline treatment and other clinical variables such as AJCC stage, tumor grade, node status, receptor status, etc. may confound the iGenSig models. Among anthracyclines, epirubicin is preferred for treating ER-negative breast cancer patients and has the best GDSC iGenSig model. We thus used the iGenSig model for epirubicin as a predictor for the anthracycline treatment. The interactions of the paclitaxel model with these confounding variables are assessed using logistic regression (see Methods). Our result showed that the most confounding variable appear to be AJCC stage (*p* = 0.048), followed by the Epirubicin model (*p* = 0.069) and tumor grade (*p* = 0.093) (Fig. 6e). When stratified by the AJCC stage, both the Lapatinib model and the Epirubicin model showed significant predictive values in stage I-II tumors, which were diminished in stage III tumors (Fig. 6f).

To verify this result, we examined another neoadjuvant clinical study carried out in a Japanese breast cancer patient cohort at Osaka University Hospital (OUH) testing neoadjuvant paclitaxel followed by 5-fluorouracil, epirubicin, and cyclophosphamide (P-FEC)^30^. This study included both ER-positive and negative patients, and only pCR is provided as a clinical endpoint. Consistently, the paclitaxel model showed significant predictive value on pCR in the stage I-II breast cancer patients only (*p* = 0.0004). Within the Stage I-II patient group, the iGenSig models for paclitaxel, 5-fluorouracil, and epirubicin showed an AUROC of 0.74, 0.71, and 0.67 respectively, and the composite predictive models based on all three models achieved an AUROC of 0.81. Taken together, these data suggest that in combination therapy, the iGenSig models derived from single-drug treatment data can be confounded by the therapeutic benefits derived from other drugs in the combination, as well as confounding drug resistance factors, such as ER overexpression and late tumor stage. Stratifying the patients based on known confounding factors and combining the models for different drugs included in the regimen will help better observe the predictive effect of the iGenSig models on patient response to combination drug therapies.

### Comparing the iGenSig algorithm with standard machine learning algorithms on modeling drug responses

**Fig. 7:**
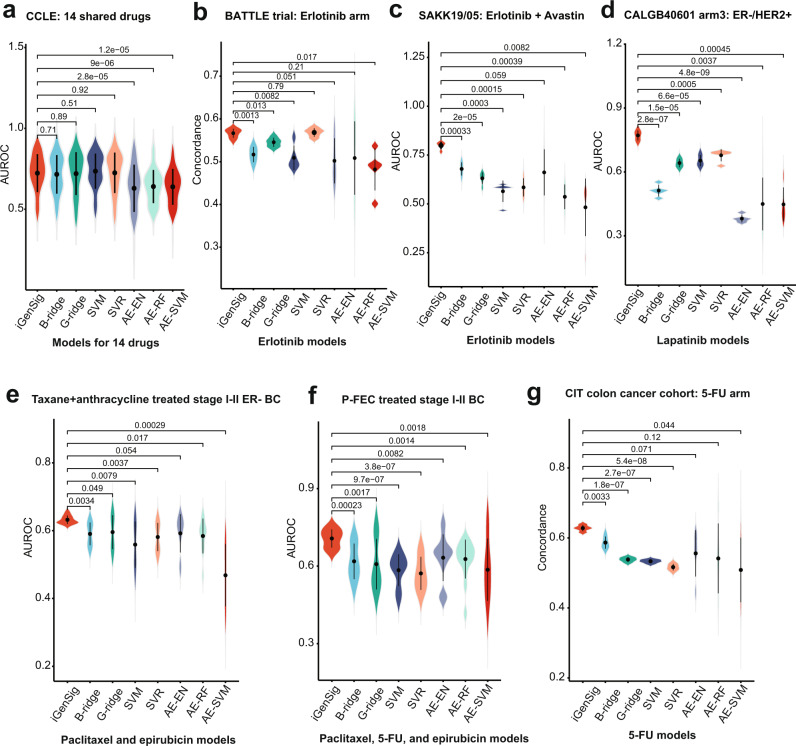
Comparisons between the iGenSig algorithm and standard machine learning algorithms on modeling drug responses. **a** The Prediction performance of the iGenSig algorithm and machine learning methods on the CCLE validation dataset for 14 drugs. If a drug is profiled by both GDSC batch 1 and 2, the drug sensitivity data from batch 1 are used in the analysis. **b**–**g** The Prediction performance of the iGenSig algorithm and machine learning methods on US BATTLE trial (**b**), Swiss SAKK 19/05 trial (**c**), CALGB40601 trial (**d**), neoadjuvant taxane-anthracycline study (**e**), neoadjuvant P-FEC study (**f**), and the French CIT colon cancer clinical study (**g**). The predictive models for the respective drugs annotated under each plot were generated based on 80% cell lines from GDSC with five permutated training sets. For ML methods, the supervised learning was directly performed on the original high-dimensional genomic features using Gaussian family ridge regression (G-ridge) and support vector regression (SVR) based on drug sensitivity measurements or using Binary family ridge regression (B-ridge) and support vector machine (SVM) based on binary sensitivity labels. For AI methods, the unsupervised learning was performed by autoencoder (AE) and supervised learning was performed using various machine learning tools including elastic net (EN), random forest (RF), and support vector machine (SVM) based on binary sensitivity labels. The *p* values are based on two-sided student’s *t*-tests. The AUROC for predicting patient binary responses (pCR for breast cancer clinical studies or objective response for the SAKK 19/05 trial) or the concordance of the predictive scores with patient survival (PFS for the BATTLE trial and OS for the CIT cohort) are shown in the figure depending on the best available clinical endpoints for these studies. The mean and standard deviations are shown in the violin plots.

Next, we sought to compare the performance of the iGenSig algorithm with standard machine learning or deep learning algorithms. First, we compared the iGenSig algorithm with the ridge regression and Support Vector Regression (SVR), which are the limited available machine learning algorithms capable of carrying out predictive modeling using ultra-high-dimensional features. In addition, we also performed ridge regression and Support Vector Machine (SVM) modeling based on binomial drug sensitivity labels. For AI-based methods, we computed the unsupervised representation of the genomic features for dimensionality reduction based on the autoencoder deep learning method, as previously reported^31,32^. The resulting synthetic features were then fed to the machine learning methods for supervised learning on drug responses, such as elastic net, support vector machine (SVM), or Random Forest (RF) (Supplementary Fig. 6). We then applied these algorithms to model cancer cell sensitivity to the fourteen drugs shared by GDSC and CCLE datasets, and to model patient responses in the six clinical trial datasets, including BATTLE, SAKK 19/05, French CIT, CALGB40601, neoadjuvant taxane-anthracycline, and P-FEC studies. Our results showed that iGenSig models have the highest overall performance on clinical trial datasets among all modeling methods (Fig. 7). Ridge regression and SVR/SVM models worked better than AI-based models and are comparable to the iGenSig models on the CCLE validation dataset (Fig. 7a). This suggests that dimensionality reductions during AI-based modeling may reduce the cross-dataset prediction performance. The better performance of iGenSig models on clinical trial datasets supports the utility of our algorithm design in improving cross-dataset modeling for patient subjects via leveraging the redundancy within the integral genomic signature and extracting unbiased genomic information from unlabeled large tumor cohorts (Fig. 7b–g). More important, iGenSig is a white-box algorithm that has the much-needed transparency required for clinical application compared to the machine learning algorithms based on complex mathematical systems, which allows for easier interpretation of the biological mechanisms underlying the models.

Moreover, we also compared iGenSig with machine learning algorithms on a PDX genomic dataset that measured the sensitivity of Pan-cancer PDX tumors against 36 single drugs^33^. These include nine GDSC profiled drugs, seven of which have more than one PDX responder in this dataset. Our results show that the GDSC models for these seven drugs showed diverse predictive values on these PDX models so all modeling methods showed large deviations in predictive results for different drugs (Supplementary Fig. 7). The iGenSig, ridge regression, and SVR/SVM models showed comparable predictive performances, all of which are better than AI-based methods. We speculate that the observed large deviations could be in part attributed to the tumor microenvironment factors important for some drugs that cannot be modeled using in vitro cultured cell lines (as discussed below) or the lack of responders in this dataset.

## Discussion

Here we introduce a class of white-box methods called integral genomic signature analyses that leverage the high-dimensional redundant genomic features as an integral genomic signature to enhance the transferability of multi-omics-based modeling for precision oncology, a concept like the use of redundant steel rods to reinforce the pillars of a building (Fig. 1b). The iGenSig method is designed to address the transparency, cross-dataset applicability, and interpretability issues for big data-based modeling. The iGenSig models demonstrated improved performances in cross-applicability to clinical trial datasets, tolerating the experimental variations and bias in the genomic data. IGenSig models can be managed in every detailed step, and the underlying pathways can be readily biologically interpreted through the concept signature enrichment analysis we developed^9^. The performance of iGenSig models appears to at least in part depend on the availability of significant genomic correlates, which provided insights into the different performances of iGenSig models on different drugs. We expect that iGenSig as a class of big data-based modeling methods will have broad application in modeling therapeutic responses based on pharmacogenomics and clinical trial datasets. Further, iGenSig can also be potentially applied to predict other cancer behaviors to facilitate clinical decisions such as the aggressiveness of carcinoma in situ, or metastatic potential of clinically localized tumors.

It is interesting to note that our iGenSig model for Erlotinib demonstrated predictive value on both preclinical tests on patient-derived xenografts and two clinical trials on human subjects, including the US BATTLE trial and the Swiss SAKK 19/05 trial. Interpretation of this model revealed that upregulation of MYC target gene signature and downregulation of ZEB1 target genes are characteristic of Erlotinib resistance signature, whereas induction of EMT is associated with reduced but intermediate responses. While both EMT and ZEB1 has been found to medicate acquired resistance to EGFR inhibitors in non-small cell lung cancer^34,35^, our iGenSig signature suggested the discordance of ZEB1 overexpression with EMT induction and their differential contribution to Erlotinib resistance in Pan-cancer cell lines. This implies that the phenotypic changes other than EMT induced by ZEB1 may contribute to Erlotinib resistance at the Pan-cancer scale. Consistent with this finding, ZEB1 has been reported to exert more critical functional consequences than EMT itself. For example, it has been proposed that specific EMT inducers such as ZEB1, but not the EMT state, determine cancer stem cell properties^36^. Future experimental studies will be required to determine the differential contribution of ZEB1 and EMT to Erlotinib resistance in human cancers.

On the other hand, our GDSC iGenSig model for sorafenib failed to predict patient response in the sorafenib treatment arm in the BATTLE trial dataset. To explore if this is attributable to the differences in the in vitro and in vivo microenvironments, we examined the primary targets of this drug. Sorafenib is a multi-kinase inhibitor of Raf-1, B-Raf, and VEGFR-2. Among these, VEGFR-2 is a VEGF receptor involved in angiogenesis. In light of this, we wonder if the iGenSig models based on in vitro cell line responses failed to model in vivo tumor responses to VEGFR inhibition. Based on the literature, VEGFA amplification is a known biomarker for Sorafenib response^37^, whereas CXCL8 (IL8) are known to induce VEGF overexpression in endothelial cells and promote angiogenesis^38^. Correlating these biomarkers with the iGenSig scores revealed that in the Sorafenib treated arm of the BATTLE trial, the sensitive tumors with low iGenSig scores appear to overexpress VEGFA and CXCL8, as well as the AMP-activated protein kinases (PRKAA1/2) important for stimulating VEGF expression and angiogenesis^39^ (Supplementary Fig. 8). This suggests that the inability of the GDSC iGenSig model to predict patient response to sorafenib in the BATTLE trial may be attributed to the anti-angiogenesis activity of sorafenib, which cannot be modeled using in vitro cultured cell lines. This reflects the limitation of modeling patient tumor response based on in vitro cell line models.

Moreover, it is interesting to note that the iGenSig scores for the BATTLE and SAKK 10/05 trials appear to accentuate different signature pathways. The iGenSig scores for the BATTLE trial accentuate the epithelial signature on tumors with high iGenSig scores, whereas the iGenSig scores for the SAKK 10/05 trial accentuate MYC target signature on tumors with low iGenSig scores (Fig. 4d). This could be due to the inability of the current iGenSig modeling method to factorize these signature pathways and model their interactions, or due to the confounding effect of different immunological contextures in these tumors. Future studies will be required to examine the effect of immune cell infiltrates on bulk sequencing-based modeling, as well as develop new iGenSig methods that can factorize the different pathway signatures as predictive pillars, and better model their predictive interactions on treatment outcomes.

Our analyses of six clinical trial datasets suggest that the iGenSig models for single-drug treatment show better predictive values in patients treated with the respective monotherapy. The major challenges to apply these single-drug models to combination therapies include the therapeutic benefits derived from the other drugs and known resistance factors such as ER overexpression and advanced tumor stage III. These confounding variables cannot be modeled based on the GDSC cell line panel. First ER-positive breast cancer cells typically do not grow in vitro thus there are very limited ER-positive cell line models in the GDSC cell line panel. Second, stage III breast tumors are characterized by extensive lymph node involvement (≥4 nodes) or chest wall or skin invasion. Such regional metastasis may create a tumor microenvironment leading to drug resistance, which cannot be recreated when the cell lines are generated from primary tumors.

The remaining issue to be addressed for iGenSig modeling is how to eliminate the effect of confounding genomic features resulting from the imbalanced distribution of confounding factors such as gender or prognostic factors that can impact patient outcomes such as metastasis, etc. While this issue may be less impactful in modeling cell line responses when a large number of cell line subjects are included, it could become more consequential when a smaller number of subjects are tested in the clinical trial. In this case, the genomic features associated with the confounding factor may be identified and excluded from the iGenSig model through multivariate statistics. In addition, the confounding clinical variables that affect prognosis such as local or distant metastasis should be accounted for via multivariate statistics or stratification methods when assessing the performance of iGenSig models in predicting patient survival outcomes. This could be particularly helpful for modeling the clinical trials testing combination drug therapies where the predictive powers of the iGenSig models derived from single-drug treatment are relatively weak due to the therapeutic benefits from combinatory drugs. Future studies will be required to further optimize the iGenSig methods for modeling clinical trial datasets and taking into consideration of these biological variables and confounding factors.

## Methods

### Data retrieval

The drug response data, gene expression data, and mutation data are from the Genomics of Drug Sensitivity in Cancer Project (GDSC), and the Cancer Cell Line Encyclopedia (CCLE) as of September 2018. The GDSC and CCLE gene expression data were retrieved from ArrayExpress (E-MTAB-783) and NCBI GEO (GSE36133), respectively and normalized using Robust Multi-Array Averaging (RMA)^40^. Drug sensitivity data, mutation data, and cell line annotations were downloaded from the GDSC (http://www.cancerrxgene.org/downloads) or CCLE (http://www.broadinstitute.org/ccle) websites. The newly released batch 2 drug sensitivity dataset are downloaded from the GDSC website as of May 2021. The TCGA Pan-cancer gene expression and mutation datasets were retrieved from the UCSC Xena browser (https://xenabrowser.net/datapages/). The gene expression and mutation data for the PDX tumors were retrieved from the supplementary dataset of the original publication^33^. The clinical trial datasets are retrieved from Gene Expression Omnibus (GEO, https://www.ncbi.nlm.nih.gov/geo) or the database of Genotypes and Phenotypes (dbGaP, https://dbgap.ncbi.nlm.nih.gov/), and the detailed access information are provided in the Data Availability section. All the genomic datasets included in this study are summarized in Supplementary Table 1.

### Extracting genome-wide expression and mutation features for cell line and tumors

Based on gene expression and somatic point mutation datasets, we extracted genome-wide differential gene expression (DGE) and mutation features and generated an integrated genomic feature file. For gene expression datasets, quantile normalizations were performed and genes with standard deviations of less than 20% percentile are filtered. We then calculated log2 transformed fold changes of the expression values compared to the trimmed mean of expression values (excluding the 10% largest and 10% smallest values). To eliminate zero values during log2 transformation, we added 1 to the expression value across all cell lines or tumors. Based on the mean and standard deviation (SD) of fold changes, we assigned the cell lines or tumors into the following overlapping groups: “Up_Level1” group with the fold change above Mean + 1 × SD for a given gene; “Up_Level2” group with the fold change above Mean + 2 × SD; …, and “Up_Level6” group with the fold change above Mean + 6 × SD Likewise, “Down_Level1”, “Down_Level2”, … and “Down_Level6” grouped cell lines based on Mean − 1 × SD, Mean − 2 × SD, and Mean − 6 × SD. The 12 “Levels” were labeled as genotypic features for each given gene and the binary genomic features are compiled as a genomic matrix transposed (GMT) file format. Similarly, we extracted binary genomic features to represent point mutations. The mutation hotspots and nonsynonymous somatic mutations such as missense, nonsense, and frameshift are assigned as mutation features. Each recurrent mutation hotspot and each recurrently mutated gene were assigned as separate features.

### Defining drug responses of cancer cell lines

Drug responses of cancer cell lines are represented by the area under the dose-response curve (AUC) in GDSC or the area over the dose-response curve (Act Area) in CCLE^11,41^. We first tested the skewness of the AUC measurements for each drug in the GDSC dataset. A negative skewness distribution indicates that the drug has high AUC measurements (lack of responses) in most of the cell lines, but low AUC measurements (sensitive responses) in a small subset of the cell lines, and a lower level of skewness indicates a higher level of outstanding responses. To ensure the drugs have sufficient outstanding responders for training and testing the algorithm, the GDSC drugs with negative skewness and more than 20 sensitive cell line subjects are included in our iGenSig cancer cell line modeling. We then defined sensitive drug responses of cell lines based on Act Areas using the standard waterfall method described in the CCLE study (implemented in the “define.response.AUC” and “define.response.ActArea” function of the iGenSig R package)^11^. The Act Area measurements for CCLE or GDSC cell lines for a given compound are sorted in ascending order to generate a waterfall distribution. The cut-off for defining sensitive subjects was determined as the maximal distance to a line drawn between the start and endpoints of the distribution. The cut-off for non-responders was determined as “median of Act Area - median absolute deviation (MAD).” The cell lines with Act Area above the sensitivity cut-off were labeled as drug-sensitive and below the resistance cut-off were labeled as drug-resistant. The cell lines with Act Areas between the cut-offs for drug sensitivity and resistance were labeled as intermediate.

### Calculating feature weights and selecting significant genomic features

To define the weight (${\omega }_{i}$) of each genomic feature in predicting sensitive drug responses, we leveraged the weighted Kolmogorov–Smirnov (WKS) statistics^10^ to test the enrichment of the feature-positive cell line in the cell line panel sorted in descending order based on Act Area (Fig. 1). The enrichment score (*ES*) for each genomic feature is calculated using the same algorithm as that implemented in Gene set enrichment analysis (GSEA)^10^. To prevent bias, we excluded the genomic features defining fewer than 5 cell lines during the calculation of GenSig scores. Likewise, we calculated the weights for each genomic feature in predicting resistant drug responses based on the cell line panel sorted by AUC in descending order. To prevent overfitting, for a given cell line *x* in the training set, cell line *x* is excluded from calculating the ES scores for the genomic features associated with cell line *x*. We assessed the significance of the observed ES by comparing that to the random ES scores calculated by random features with the same numbers of positive cell lines. Repeat this step until 2000 random enrichment scores were calculated, then the normalized enrichment score (NES) was calculated by:

1 $${{{{{{\mathrm{NES}}}}}}}={{{{{{\mathrm{ES}}}}}}}/{{{{{{\mathrm{mean}}}}}}}({{{{{{{\mathrm{ES}}}}}}}}_{{{{{{{\mathrm{random}}}}}}}})$$

The *p* values were determined based on the chance of random ES scores to be above the observed ES score for feature *i*, and the false discovery rate (FDR) were calculated using the R package “qvalue” for multiple comparisons. In this study, we used a universal FDR q value cutoff of 0.1 to select significant genomic features for calculating iGenSig scores. This parameter can be tuned for different drugs to further refine the model as the signal-to-noise levels of these predictive genomic features could be different for different drugs. Furthermore, we observed that some of the genes have both upregulation and downregulation features ranked as significant for predicting the sensitive drug response. We thus filtered the genes that have both upregulated features with FDR <0.1, and downregulated features with FDR <0.3, and the genes that have both downregulated features with FDR <0.1, and upregulated features with FDR <0.3. On the other hand, some of the genes have only level-1 DGE features selected as significant based on FDR <0.1, but none of their corresponding high levels of DGE features have an FDR more than 0.3 even if they define more than ten cell lines. These genomic features represent noises and are thus filtered as well.

### The algorithms for penalizing feature redundancy and methods for iGenSig modeling

To prevent the inflation of iGenSig scores from feature redundancy, we leveraged the TCGA Pan-Cancer RNAseq and exome datasets to assess the co-occurrence between genomic features associated with each cell line and generated the cosine similarity matrix of genomic features based on Otsuka–Ochiai coefficient between these features (K_ij_). We then performed clustering of the cosine similarity matrix based on Ward’s method (D2) using the R module “hClust”. The correlated feature groups are then determined based on an adaptive dynamic cluster detection method^42^, using the parameters: dynamic.method = “hybrid”, cutTree.depth = 2, and minClusterSize = 40. We then introduced a penalization factor (*ε*) which is calculated for each genomic feature *i* based on the similarity indices of the colinear genomic features associated with a given cell line _*x*_ and of the same cluster as a feature *i*:

2 $${\varepsilon }_{i}=\mathop{\sum}\limits_{j\in {{{{{{{\mathrm{Cluster}}}}}}}}_{i}}{K}_{{ij}}$$

Where K_ij is_ the Otsuka–Ochiai coefficient between feature *i* and a given genomic feature *j* from the same cluster group as a feature *i* associated with cell line _*x*._ To eliminate the cumulative effect of small overlaps between genomic features, the Otsuka–Ochiai coefficients were adjusted to 0 if K_ij_ < 0.1. Here *ε*_*i*_ is an estimator of redundancy among the genomic features of the same cluster group associated with cell line _*x*_. The penalization factor ranges from 1 (all genotypes are completely different from each other) to *n* (all genotypes are the same). We then penalized the weight *ω*_*i*_ using *ε*_*i*_, resulting in effective weight (EW):

3 $${{{{{{{\mathrm{EW}}}}}}}}_{i}=\frac{{\omega }_{i}}{{\varepsilon }_{i}}$$

The trimmed mean of *ε*_*i*_ (trim = 0.3) was then used to calculate the effective feature number (EFN):

4 $${{{{{{{\mathrm{EFN}}}}}}}}_{i}=\frac{n}{{\bar{\varepsilon }}_{T}}$$

Finally, the iGenSig score of the given cell line_x_ is computed as:

5 $${i{{{{{{\mathrm{GenSig}}}}}}}}_{\left|{{{{{{{\mathrm{Cell}}}}}}\; {{{{{\mathrm{Line}}}}}}}}_{x}\right.}=\frac{{\sum }_{i=1}^{n}{{{{{{{\mathrm{EW}}}}}}}}_{i}}{{{{{{{{\mathrm{EFN}}}}}}}}_{i}}=\frac{{\sum }_{i=1}^{n}{I}_{\{i{{{{{\rm{\epsilon }}}}}}x\}}\frac{{\omega }_{i}}{{\varepsilon }_{i}}}{\frac{n}{{\bar{\varepsilon }}_{T}}}$$

The sensitive and resistant iGenSig scores are calculated separately based on the significant genomic features selected for predicting sensitive or resistant responses. The sensitive iGenSig scores are used in this study for assessing the performance of the iGenSig models in predicting sensitive cell lines and patient subjects. Thus, the iGenSig scores labeled in the figures refer to the sensitive iGenSig scores unless otherwise noted.

When applying the iGenSig models to the validation datasets, the weights of the significant genomic features (*q* < 0.1) calculated based on GDSC datasets will be used for calculating the iGenSig scores based on the presence of these features in a patient or cell line subject in the validation dataset. The weights of these features associated with that subject were then penalized based on the feature redundancy levels assessed using the TCGA Pan-cancer dataset (for CCLE dataset) or cancer type-specific TCGA dataset (for a clinical trial dataset of specific tumor type), and the iGenSig scores were calculated using the same algorithm. Thus, here only the weights from the GDSC dataset are used for modeling the validation datasets.

### Benchmarking the performance of the iGenSig algorithm

To benchmark the performance of the iGenSig algorithm in determining drug sensitivity, we randomly selected 20% of GDSC cell lines treated by a specific drug as an internal test set. We assigned the rest of the 80% cell lines as a train set and performed this randomized sampling five times. The distributions of drug-sensitive and resistant cell lines were required to be balanced between the train and test set in each sampling. The CCLE dataset was used as an external validation set of our predictive models to assess their applicability to an independent dataset. The area under the ROC curve (AUROC) of the iGenSig scores was calculated based on the binary response of the cell lines determined based on the sensitive cutoff discussed above, and the optimal cut-points of iGenSig scores are determined using the R module “coords” of the “pROC” package. The cell line subjects were divided into sensitive cell lines and other cell lines that include both intermediate and resistant cell lines, and the sensitive iGenSig scores are used when assessing the predictive values of the iGenSig models.

To test if the iGenSig predictions rely on the genomic features of the primary drug targets, we removed the drug target gene features for Erlotinib, Lapatinib, or Nilotinib from GDSC and CCLE genomic feature sets. We then built the iGenSig models based on the genomic features devoid of drug targets and assessed their performance on the internal test set (20% of GDSC cell lines) or external validation set (100% of CCLE cell lines). To examine if excluding the hematologic cancer cell lines from the GDSC training dataset can improve the prediction performances of iGenSig models on the drug sensitivity of CCLE solid cancer cell lines, we removed leukemia, lymphoma, and myeloma cell lines from the GDSC dataset when performing the modeling for the shared 14 drugs, and then applied the models to CCLE solid cancer cell lines (Supplementary Fig. 2b).

### Meta-analyses of clinical trial and prospective clinical study datasets

To examine the predictive values of the iGenSig models on patient subjects, we compiled six clinical trial or prospective clinical study datasets. The detailed information of the clinical datasets used in this study including enrollment details, treatment arms, available endpoints, available genomic datasets, and source publications are summarized in Supplementary Table 1. The iGenSig model for the specific drug tested on a given treatment arm of the trial are developed based on the GDSC dataset, and then applied to the genomic features of the clinical trial datasets. These include the models for Lapatinib (Drug ID: 119), Erlotinib (Drug ID: 1), Sorafenib (Drug ID: 30), 5-Fluorouracil (Drug ID: 179), Paclitaxel (Drug ID: 1080), and Epirubicin (Drug ID: 1511). The uses of clinical endpoints are dependent on the clinical information provided by the authors of the original publications. Overall survival (OS) is the preferred endpoint of choice^43^, followed by pathologic complete response (pCR). For the CALGB40601 trial, pCR in the breast and axilla is used in our analysis, as no tumor residuals in both breast and lymph nodes can best discriminate patient outcomes^44^. Other endpoints will be used in the analysis if OS and pCR are not available.

### Implementation of machine learning and deep learning methods

All machine learning and deep learning methods used in this study are implemented in R. For ridge regression and support vector regression, we performed the modeling directly based on the original high-dimensional binary genomic features. For ridge regression, we implemented using the R package “glmnet”. The Gaussian family is used for drug sensitivity measurements and the Binary family is used for binary sensitivity classifications. For tuning the ridge regression model, the best “lambda.1se” was obtained from tenfold cross-validation. Support vector regression is implemented using the “svm” function of the “e1071” package using default parameters based on either drug sensitivity measurements or binomial sensitivity labels. For AI-based methods, we applied the deep learning method autoencoder^45^ to perform unsupervised representation learning for dimensionality reduction and machine learning prediction algorithms for supervised learning of therapeutic responses using the low dimensional features generated by autoencoder, as previously reported^31,32^. The models are developed based on 80% of GDSC datasets (five permutated training sets). The autoencoder model was built with three hidden layers with the unit sizes in each layer designed in accordance with a previous report^31^. We then applied the unsupervised representation of the genomic correlates to supervised learning methods including elastic net, artificial neural network, Random Forest (RF), and support vector machine (SVM) for prediction modeling. Elastic net is a regression method that combines lasso and ridge regularization with the two hyperparameters, alpha and lambda. Alpha is a mixing parameter to define the relative weight of the lasso and ridge penalization terms and lambda determines the amount of shrinkage^46^. We identified alpha with the best tuning and optimized for predictive performance over a range of lambdas. Regression was performed using the glmnet R package (ver. 4.0.2). We implemented an RF regression model using randomForest R package (ver.4.6.14). We specified 1,000 trees to grow and ensure every object gets predicted multiple times. We used SVM with the linear kernel method, “svmLinear2”, provided by the caret R package (ver. 6.0.86). We specified tuneLength = 10 in the tuning parameter grid and accuracy metric. For all modeling methods, the models were developed using the same genome-wide gene expression and mutation features we compiled, and we used the same training and external validation sets of cell lines and patient subjects as in iGenSig modeling. One model is developed for each drug based on each permutation set, which are then applied to the CCLE and clinical trial datasets. To match the genomic features, the genomic features are set to zero if they do not present in the validation sets as in the iGenSig modeling.

### Pathway enrichment analysis for integral genomic signature

To identify the pathways characteristic of the integral genomic signature for Erlotinib resistance modeled from the GDSC dataset, we first extracted the genes involved in the iGenSig signature and then classified these genes into positive contributing genes and negative contributing genes. The positive contributing genes are defined as upregulated genes or genes with hotspot mutations. The negative contributing genes are defined as downregulated genes or mutated genes without mutation hotspots. The pathways enriched in the positive or negative contributing genes for predicting Erlotinib or 5-FU sensitive responses are analyzed by the Concept Signature Enrichment Analysis (CSEA) developed in our previous study^9^ using the Hallmark pathway gene sets from MSigdb (http://www.gsea-msigdb.org). The resulting top pathways are disambiguated via correcting the crosstalk effects between pathways, to reveal independent pathway modules^47^. A *p* value <0.01 is used as a cutoff for disambiguation. The functional associations between the significant pathways are then assessed using our CSEA method that computes the enrichment of each pathway *x* on the human gene list sorted descendingly based on the universal concept signature scores of each pathway *y*^9^. The pathway network was visualized using the “igraph” R package (ver. 1.2.4.2).

### Statistical analysis

The correlation between the predictive powers of the iGenSig models (AUROCs) with the total number of significant predictive genomic features (square root transformed) for each drug was determined using Spearman’s correlation coefficient. For survival analysis, the *P* values are calculated based on log-rank tests and a data-driven cut point of high iGenSig scores was determined using the R-package “maxstat”^48^. The concordance is used as a measure of goodness-of-fit for the predictive models in Coxph survival regression, which defines the probability that the predictive scores goes in the same direction as the survival data. To estimate the percent of outcome variations that can be explained by different factors, we fitted a logistic regression model and calculated the Pseudo-R^2^ for different predictive factors and clinical variables such as iGenSig scores, clinical stage, tumor grade, etc. For interaction analysis, multiple logistic regression models are generated for pair-wise interactions of the iGenSig model with each of the confounding factors, and the multiple models are compared with the simple logistic regression model via the Chi-Square test implemented in the “anova” function. *P* values of the box or violin plots were analyzed by paired or unpaired Student’s *t*-tests. Two-sided tests are used when both directions are to be tested, such as when comparing the different modeling methods. One-sided tests are used to determine if there is a difference between groups in a specific direction such as that defined by the iGenSig scores.

### Reporting summary

Further information on research design is available in the Nature Research Reporting Summary linked to this article.

## Supplementary information

Supplementary Material

Supplementary data 1

Reporting Summary

## Data Availability

The source data used in this study can be retrieved from a public data repository and are summarized in Supplementary Table 1. Drug sensitivity data, mutation data, and cell line annotations are available through the GDSC (http://www.cancerrxgene.org/downloads) and CCLE (http://www.broadinstitute.org/ccle) websites. The TCGA Pan-cancer datasets are available through the UCSC Xena browser (https://xenabrowser.net/datapages). The publicly available microarray gene expression data for clinical trials are obtained from GEO (https://www.ncbi.nlm.nih.gov/geo). These include BATTLE trial, (GSE33072), Swiss SAKK 19/05 trial (GSE37138), a multicenter clinical study carried out by the French CIT program (GSE39582), multicenter taxane treated stage I-III basal-like breast cancer patient cohort (GSE25055 and GSE25065), and OUH neoadjuvant P-FEC study on Japanese breast cancer patients (GSE32646). The RNAseq and mutation data for the CALGB40601 clinical trial dataset are retrieved from dbGaP (phs001570.v2.p1) that are available under restricted access controlled by the NCI Data Access Committee [NCIDAC@mail.nih.gov]. A minimum dataset compendium containing the TCGA, GDSC, CCLE, BATTLE, and French CIT datasets is made available through Zenodo (https://zenodo.org/badge/latestdoi/444456261).

## Code availability

The R modules for iGenSig modeling are available through Github (https://github.com/wangxlab/iGenSig/) and through Zenodo (https://zenodo.org/badge/latestdoi/444456261)^49^.
