# Supplementary material for: An integral genomic signature approach for tailored cancer therapy using genome-wide sequencing data
Source: Nat Commun. 2022 May 26;13:2936. doi: 10.1038/s41467-022-30449-7 (PMC9135729; doi:10.1038/s41467-022-30449-7)
Supplement: Supplementary file 1 — Supplementary Material [file 41467_2022_30449_MOESM1_ESM.pdf]

SUPPLEMENTARY MATERIALS

Supplementary Figures

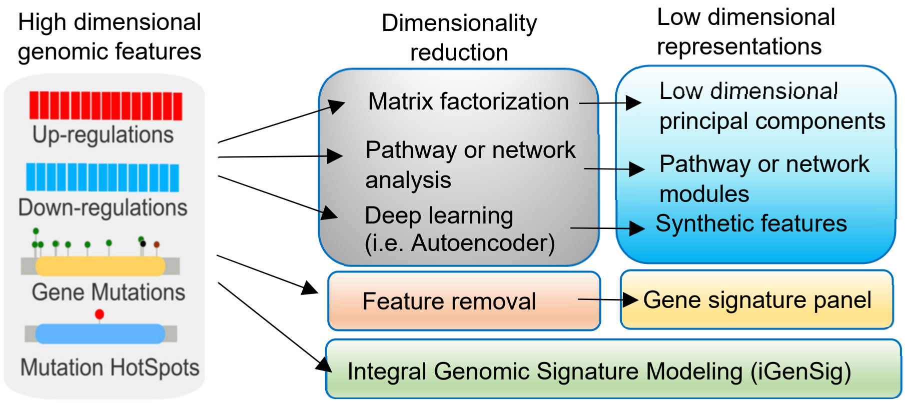

Supplementary Figure 1. Schematic showing the key difference between integral genomic signature modeling and conventional gene signature or standard AI methods in handling high-dimensional features. An integral genomic signature is defined as the comprehensive set of high-dimensional genomic features predictive of a given clinical phenotype such as therapeutic response. iGenSig represents a new class of modeling methods that directly utilize high-dimensional redundant genomic signature for predictive modeling based on multi-omics data.

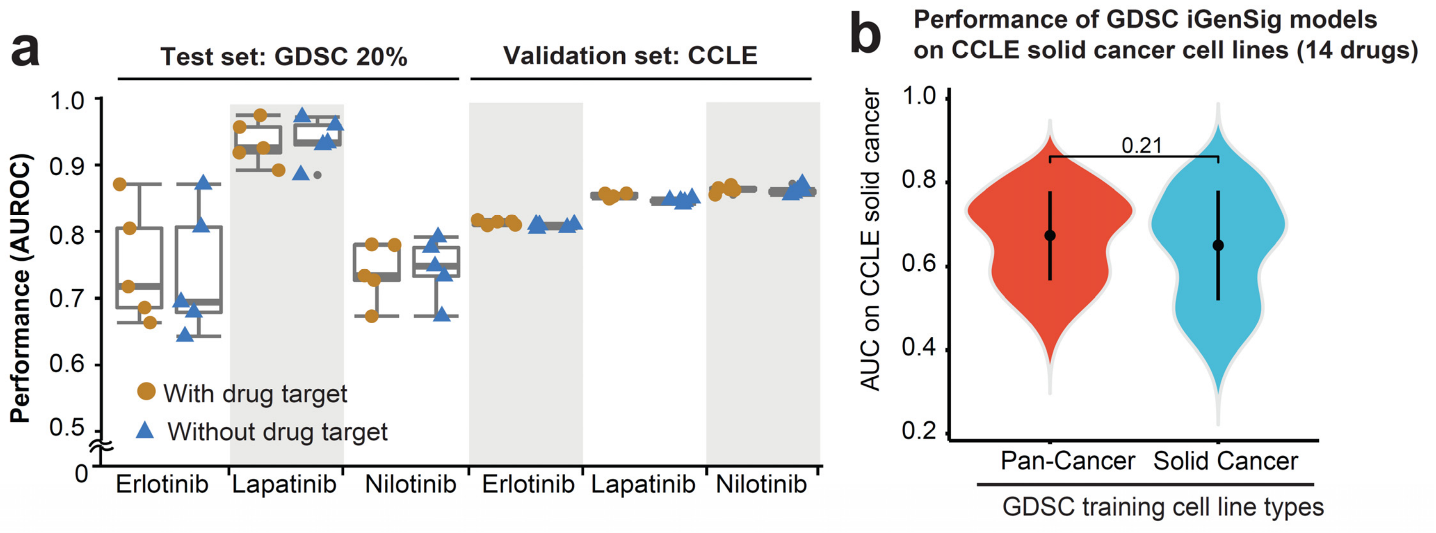

Supplementary Figure 2. The prediction performance of iGenSig models generated based on genomic features devoid of primary drug targets, or based on solid cancer cell lines. (**a**) The box plot shows the performance of the iGenSig models for Erlotinib, Lapatinib, and Nilotinib assessed on GDSC testing set (left) or CCLE validation set (right) with or without deleting the genomic features derived from the respective drug targets (Erlotinib: EGFR, Lapatinib: EGFR, ERBB2, Nilotinib: ABL1, KIT, and PDGFRB). The boxplot elements indicate the maxima, 75th percentile, median, 25th percentile, and minima. (**b**) The violin plot shows the performance of the GDSC iGenSig models for 14 drugs on the CCLE solid cancer cell lines. The GDSC iGenSig models are generated based on either Pan-cancer cell lines that include liquid cancer cell lines (leukemia, lymphoma, or myeloma), or solid cancer cell lines that exclude liquid cancer cell lines. The predictive models for the respective drugs annotated under each plot were generated based on 80% cell lines from GDSC with five permutated training sets. The mean and standard deviations are shown in the violin plots.

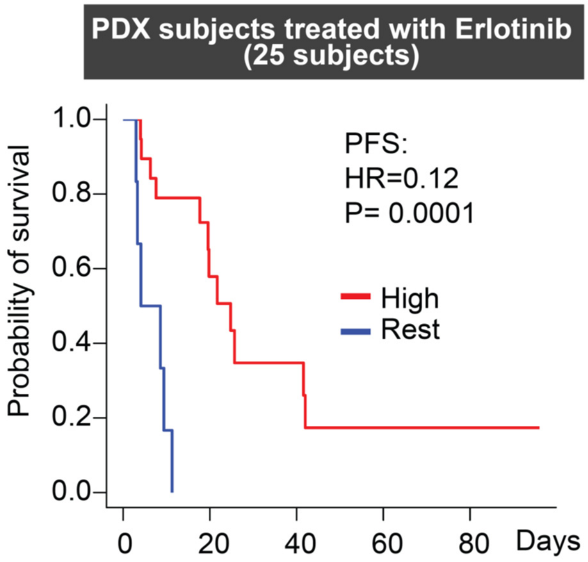

Supplementary Figure 3. Kaplan–Meier plot showing the predictive value of the GDSC iGenSig models for Erlotinib on the PDX subjects treated with Erlotinib monotherapy. The P-value is calculated based on log-rank test, and a data-driven cut point of high iGenSig scores was determined as previously described (ref. 47), using the R-package “maxstat”.

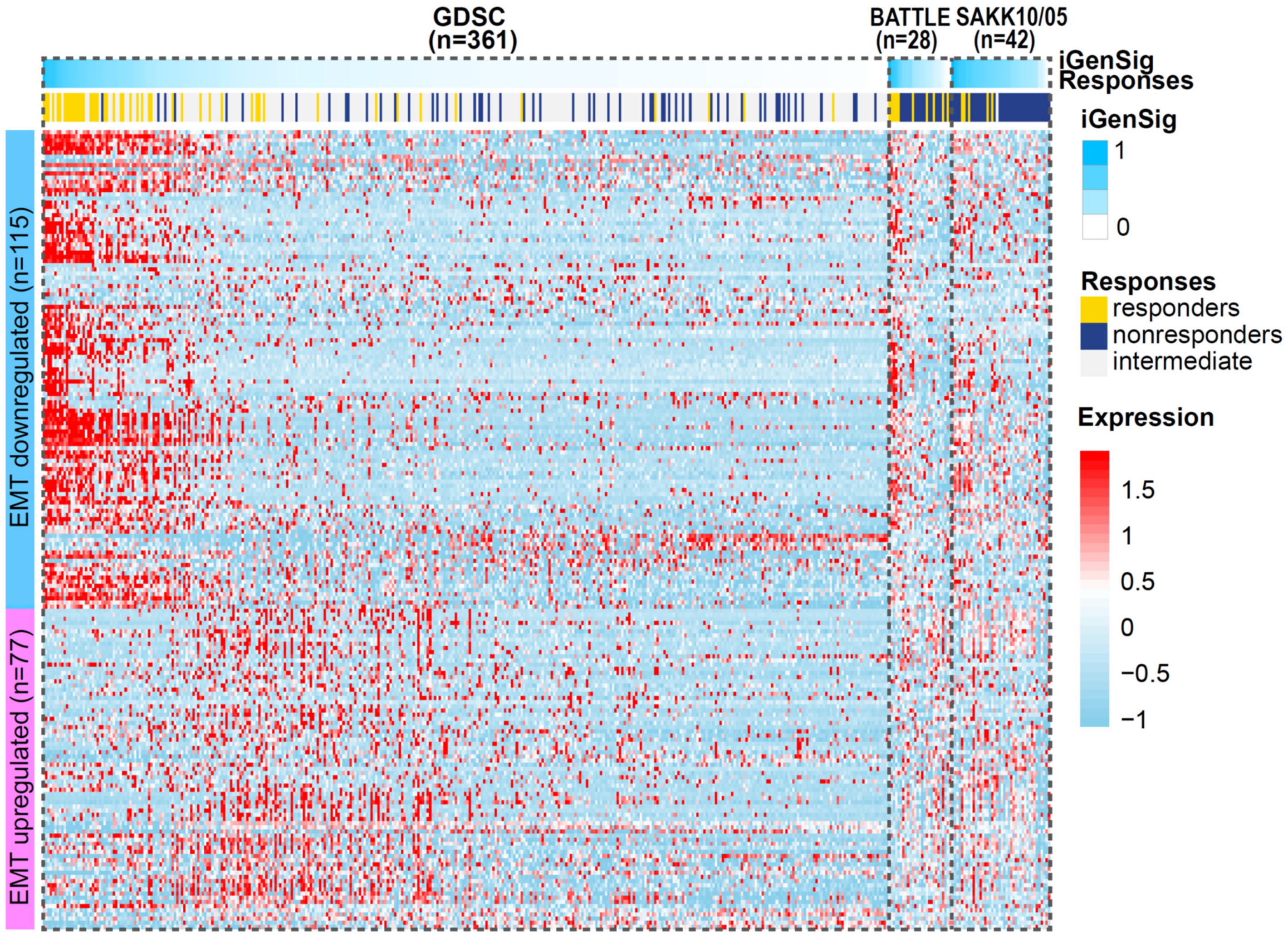

Supplementary Figure 4. The expression of upregulated or downregulated gene signature in EMT in association with the iGenSig scores for Erlotinib in GDSC cell lines and patient subjects from the BATTLE trial. The cell line and patient subjects are sorted decreasingly by their iGenSig scores.

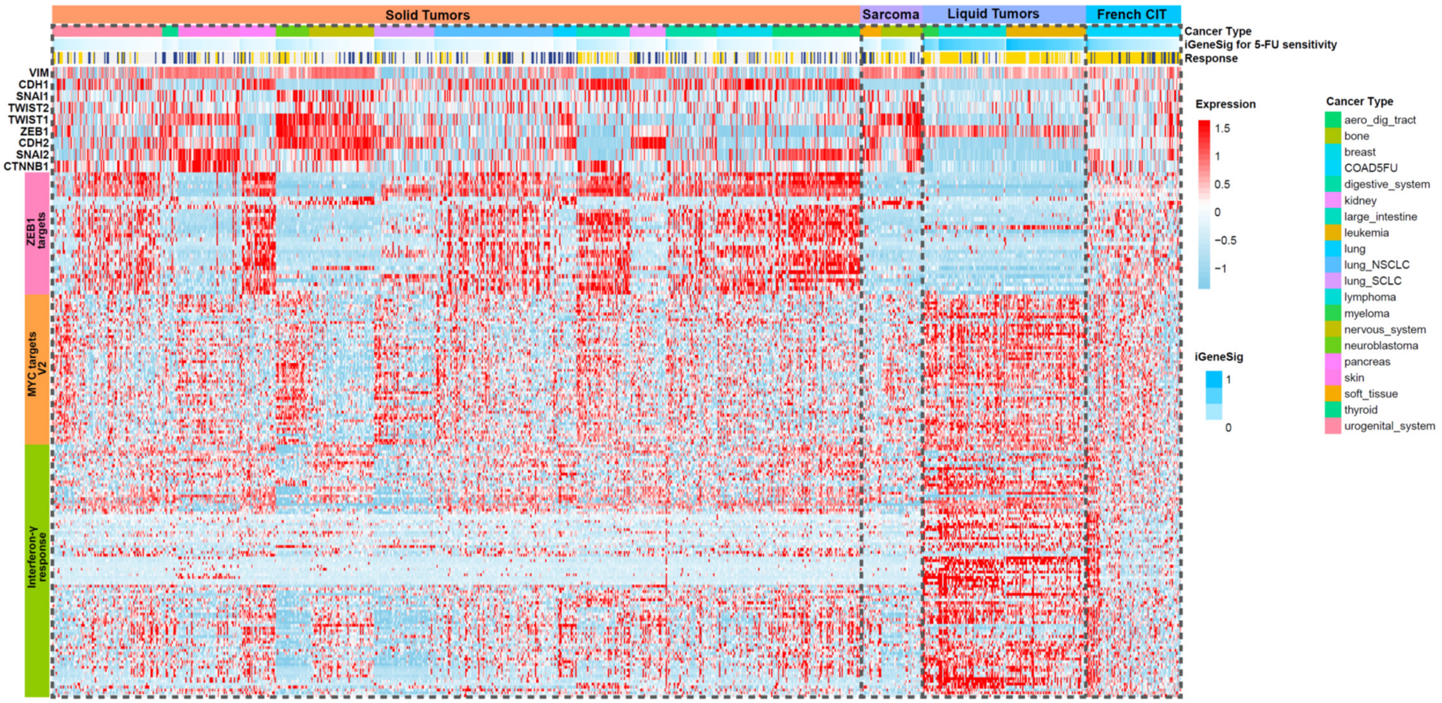

Supplementary Figure 5. Heatmap showing the associations of EMT markers and master transcription factors, ZEB1 and MYC target genes, and interferon γ responsive genes with the sensitive iGenSig scores for 5-FU in the GDSC and CIT subjects classified based on cancer types. The cell lines and patient subjects are sorted based on their cancer types and sensitive iGenSig scores for 5-FU.

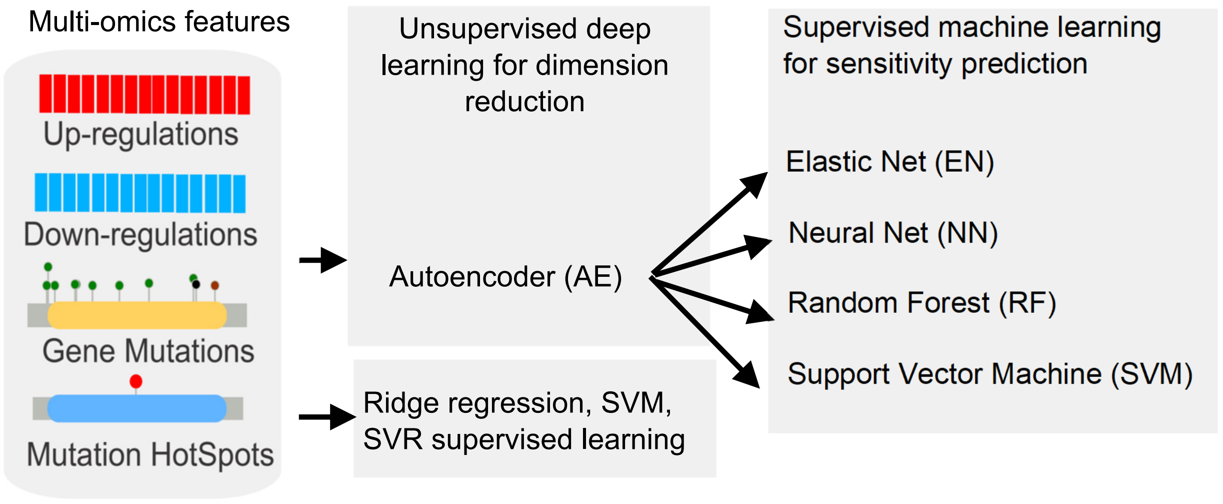

Supplementary Figure 6. Schematic showing the workflow of deep learning and machine learning methods implemented in this study for drug sensitivity prediction.

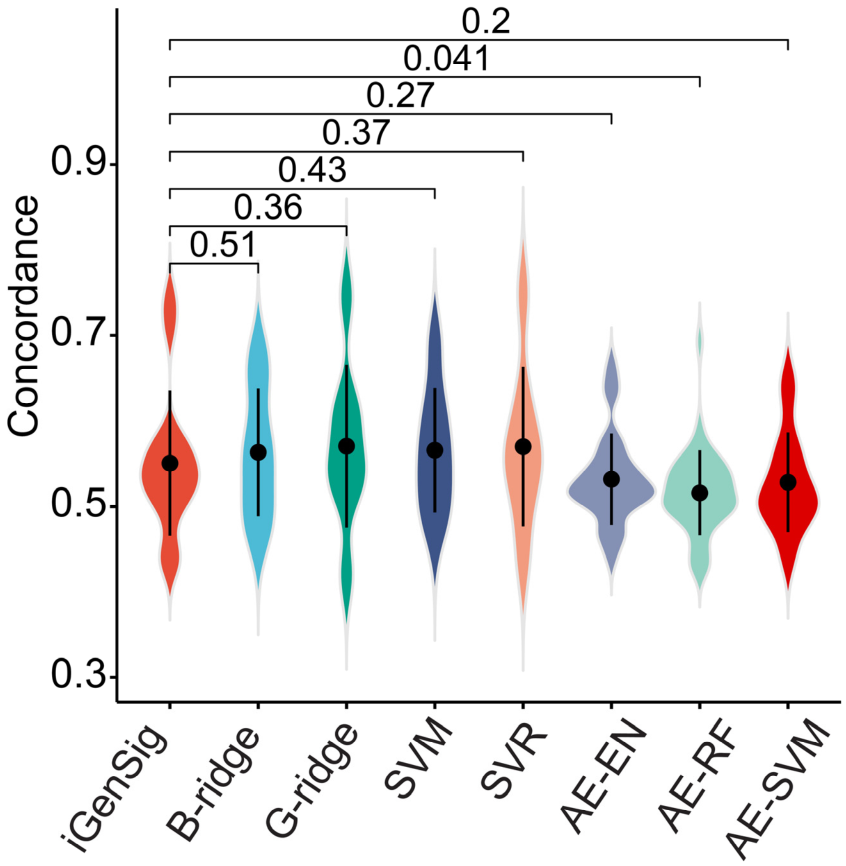

Supplementary Figure 7. Comparisons between the iGenSig algorithm and machine learning algorithms on modeling drug responses in the PDX dataset. A total of seven drugs shared between GDSC and PDX datasets have more than one responder and are shown in the figure. P-values are based on two-sided t-tests. The concordance of the predictive scores with the progression-free survival (tumor-doubling) are shown in the figure. For ML methods, the supervised learning was directly performed on the original high-dimensional genomic features using Gaussian family ridge regression (G-ridge) and support vector regression (SVR) based on drug sensitivity measurements, or using Binary family ridge regression (B-ridge) and support vector machine (SVM) based on binary sensitivity labels. For AI methods, the unsupervised learning was performed by autoencoder (AE) and supervised learning was performed using various machine learning tools including elastic net (EN), random forest (RF) and support vector machine (SVM) based on binary sensitivity labels. The predictive models for the respective drugs annotated under each plot were generated based on 80% cell lines from GDSC with five permutated training sets. The mean and standard deviations are shown in the violin plots.

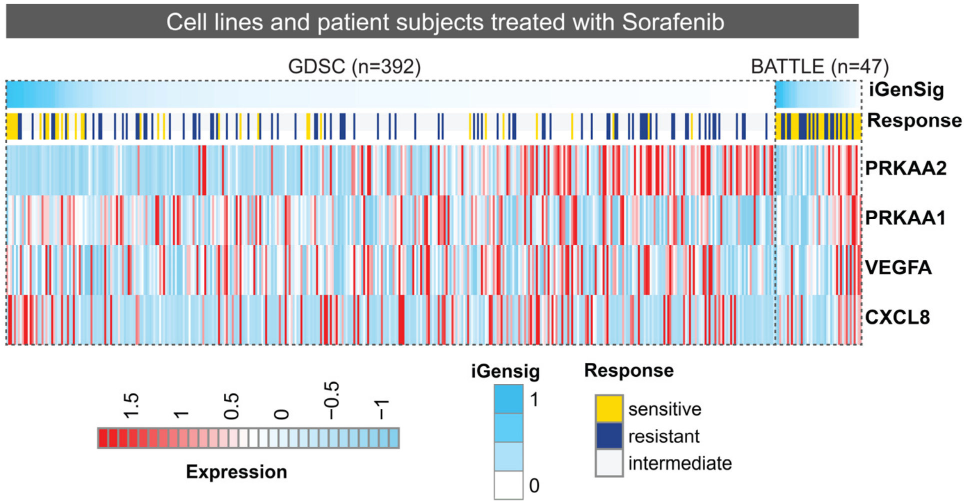

Supplementary Figure 8. The expression of VEGFA and CXCL8 in correlation with the iGenSig scores for Erlotinib in GDSC cell lines and patient subjects from BATTLE trial. The cell line and patient subjects are sorted decreasingly by their iGenSig scores.

Supplementary Tables

Supplementary Table 1. A summary of the Pharmacogenomic and clinical trial datasets used in this study.

Genomic Dataset for Drug Sensitivity (GDSC)	Pharmacologic profiles for 267 anticancer drugs across 989 cell lines.
	Affymetrix Human Genome U219 Array data
	Whole exome sequencing data
	PMID:23180760<br>Nucleic Acids Res. 2013 Jan;41(Database issue):D955-61. doi: 10.1093/nar/gks1111.
Cancer Cell Line Encyclopedia (CCLE)	Pharmacologic profiles for 24 anticancer drugs across 504 cell lines.
	Transcriptome sequencing data
	Whole exome sequencing data
	PMID: 22460905<br>Nature. 2012 Mar 28;483(7391):603-7. doi: 10.1038/nature11003
Biomarker-integrated Approaches of Targeted Therapy for Lung Cancer Elimination (BATTLE).	ClinicalTrials.gov Identifier: NCT00409968<br>Enrollment: 255 chemorefractory NSCLC patients.<br>Data source: GEO (GSE33072)<br>Treatment arms: 1) erlotinib, 2) vandetanib, 3) erlotinib + bexarotene, or 4) sorafenib.<br>Endpoints available: 8-week disease control and progression-free survival<br>Genomic data: Affymetrix Human Gene 1.0 ST Array data are available for 131 patients including patients treated with the following drugs profiled by GDSC: 1) Erolotinib (n=28), 2) Sorafanib (n=47), 3) Vandetinib (n=20), 4) erlotinib + bexarotene (n=6).<br>PMID: 23091115.<br>Clin Cancer Res. 2013 Jan 1;19(1):279-90. doi: 10.1158/1078-0432.CCR-12-1558.
The Swiss Group for Clinical Cancer Research (SAKK) 19/05 multicenter single arm trial	ClinicalTrials.gov: NCT00354549.<br>Enrollment: 103 patients with newly diagnosed or recurrent stage IIIB or IV non-squamous NSCLC. 42 patients have available lung tumor biopsy for further analysis.<br>Data source: GEO (GSE37138):<br>Single arm: combination of Erlotinib and bevacizumab (Avastin)<br>Endpoints available: objective tumor responses at 12 weeks after Erlotinib and bevacizumab treatment.<br>Genomic data: Affymetrix Human Exon 1.0 ST Array (42 lung tumor biopsies)<br>PMID: 24039832<br>PLoS One. 2013 Sep 10;8(9):e72966. doi: 10.1371/journal.pone.0072966.
The French Cartes d'Identité des Tumeurs (CIT) program	Data source: GEO (GSE39582)<br>Enrollment: patients with stage I-IV colon cancer who underwent surgery between 1987 and 2007 in seven centers.<br>Treatment arms: 5-FU monotherapy or combination chemotherapy such as FOLFIRI, FOLFOX, and FUFOL.

	Endpoints available: OS, RFS.<br>Genomic data: Affymetrix U133Plus2 gene expression data for 566 colon cancer as itemized below.<br>Treatment: 5FU FOLFIRI FOLFOX FUFOL untreated<br>Subjects (n): 82 12 23 54 411<br>PMID: 23700391<br>PLoS Med. 2013;10(5):e1001453. doi: 10.1371/journal.pmed.1001453.
CALGB 40601	ClinicalTrials.gov ID: NCT00770809<br>Data source: dbGaP (phs001570.v2.p1)<br>Treatment arms: Arm1: THL (n=108); Arm2: TH (n=109); Arm3: TL (n=60)<br>Endpoints available: pCR breast and pCR breast axilla<br>Genomic data-- RNAseq: THL (n=108); Arm2: TH (n=109); Arm3: TL (n=60)<br>PMID: 26527775<br>J Clin Oncol . 2016 Feb 20;34(6):542-9. doi: 10.1200/JCO.2015.62.1268.
Multicenter study for taxane and anthracycline–based regimens in HER2 negative breast cancer	Prospective multicenter study conducted from June 2000 to March 2010 at the M. D. Anderson Cancer Center.<br>Enrollment: Patients were those with newly diagnosed with stage I-III Her2 negative breast cancer treated with sequential taxane and anthracycline-based regimens.<br>Endpoints available: pCR, DRFS.<br>Data source: GEO (GSE25055 and GSE25065)<br>Genomic data: Affymetrix Human Genome U133A Array data for 508 cases. Patients with basal like TNBC tumors are included in our analysis.<br>PMID: 21558518<br>JAMA. 2011 May 11;305(18):1873-81. doi: 10.1001/jama.2011.593.
OUH neoadjuvant P-FEC study on Japanese breast cancer patients	Neoadjuvant clinical study carried out in a Japanese breast cancer patient cohort at Osaka University Hospital (OUH) testing neoadjuvant paclitaxel followed by 5-fluorouracil, epirubicin, and cyclophosphamide (P-FEC).<br>Enrollment: T1-4b N0-1 M0 ER positive and negative breast cancer patients<br>Endpoints available: pCR<br>Data source: GEO (GSE32646)<br>Genomic data: Affymetrix U133Plus2 gene expression data for 115 cases.<br>PMID: 22320227<br>Cancer Sci. 2012 May;103(5):913-20. doi: 10.1111/j.1349-7006.2012.02231.x.
Pharmacogenomics Data for Patient Derived Xenografts	1,000 patient-derived tumor xenograft models (PDXs)
	62 treatment arms across six indications<br>9 treatment arms are based on GDSC profiled drugs, among which 7 treatment arms have more than one responder.
	Transcriptome sequencing data
	Whole exome sequencing data
	PMID: 26479923

	Nat Med. 2015 Nov;21(11):1318-25. doi: 10.1038/nm.3954.
