# Supplementary material for: An integral genomic signature approach for tailored cancer therapy using genome-wide sequencing data
Source: Nat Commun. 2022 May 26;13:2936. doi: 10.1038/s41467-022-30449-7 (PMC9135729; doi:10.1038/s41467-022-30449-7)
Supplement: Supplementary file 3 — Reporting Summary [file 41467_2022_30449_MOESM3_ESM.pdf]

nature portfolio

Corresponding author(s): Xiaosong Wang

Last updated by author(s): Apr 9, 2021

Reporting Summary

Nature Portfolio wishes to improve the reproducibility of the work that we publish. This form provides structure for consistency and transparency in reporting. For further information on Nature Portfolio policies, see our Editorial Policies and the Editorial Policy Checklist.

Statistics

For all statistical analyses, confirm that the following items are present in the figure legend, table legend, main text, or Methods section.

n/a	Confirmed	
☐	☒	The exact sample size (*n*) for each experimental group/condition, given as a discrete number and unit of measurement
☒	☐	A statement on whether measurements were taken from distinct samples or whether the same sample was measured repeatedly
☐	☒	The statistical test(s) used AND whether they are one- or two-sided<br>*Only common tests should be described solely by name; describe more complex techniques in the Methods section.*
☐	☒	A description of all covariates tested
☐	☒	A description of any assumptions or corrections, such as tests of normality and adjustment for multiple comparisons
☐	☒	A full description of the statistical parameters including central tendency (e.g. means) or other basic estimates (e.g. regression coefficient) AND variation (e.g. standard deviation) or associated estimates of uncertainty (e.g. confidence intervals)
☐	☒	For null hypothesis testing, the test statistic (e.g. *F*, *t*, *r*) with confidence intervals, effect sizes, degrees of freedom and *P* value noted<br>*Give P values as exact values whenever suitable.*
☒	☐	For Bayesian analysis, information on the choice of priors and Markov chain Monte Carlo settings
☒	☐	For hierarchical and complex designs, identification of the appropriate level for tests and full reporting of outcomes
☐	☒	Estimates of effect sizes (e.g. Cohen's *d*, Pearson's *r*), indicating how they were calculated

Our web collection on statistics for biologists contains articles on many of the points above.

Software and code

Policy information about availability of computer code

Data collection	The drug response data, gene expression data, and mutation data are from the Genomics of Drug Sensitivity in Cancer Project (GDSC), and the Cancer Cell Line Encyclopedia (CCLE). The gene expression data for clinical trials are obtained from Gene Expression Omnibus (GEO) or dbGaP which are summarized in Supplementary Table 1.
Data analysis	The R modules for iGenSig modeling are available through: https://github.com/wangxlab/iGenSig/ and through Zenodo[https://zenodo.org/badge/latestdoi/444456261]. The iGenSig was built on R version 4.1.2.

For manuscripts utilizing custom algorithms or software that are central to the research but not yet described in published literature, software must be made available to editors and reviewers. We strongly encourage code deposition in a community repository (e.g. GitHub). See the Nature Portfolio guidelines for submitting code & software for further information.

Data

Policy information about availability of data

All manuscripts must include a data availability statement. This statement should provide the following information, where applicable:

- Accession codes, unique identifiers, or web links for publicly available datasets
- A description of any restrictions on data availability
- For clinical datasets or third party data, please ensure that the statement adheres to our policy

The source data used in this study can be retrieved from public data repository and are summarized in Supplementary Table 1. Drug sensitivity data, mutation data, and cell line annotations are available through the GDSC[http://www.cancerrxgene.org/downloads] and CCLE [http://www.broadinstitute.org/ccle] websites. The TCGA Pan-cancer datasets are available through UCSC Xena browser [https://xenabrowser.net/datapages]. The publicly available microarray gene expression data for clinical trials are obtained from GEO[https://www.ncbi.nlm.nih.gov/geo]. These include BATTLE trial, (GSE33072[https://www.ncbi.nlm.nih.gov/geo/query/

acc.cgi?acc=GSE33072]), Swiss SAKK 19/05 trial (GSE37138[https://www.ncbi.nlm.nih.gov/geo/query/acc.cgi?acc=GSE37138]), multi-center clinical study carried out by the French CIT program (GSE39582[https://www.ncbi.nlm.nih.gov/geo/query/acc.cgi?acc=GSE39582]), multi-center taxane treated stage I-III basal-like breast cancer patient cohort (GSE25055[https://www.ncbi.nlm.nih.gov/geo/query/acc.cgi?acc=GSE25055] and GSE25065[https://www.ncbi.nlm.nih.gov/geo/query/acc.cgi?acc=GSE25065]), and OUH neoadjuvant P-FEC study on Japanese breast cancer patients (GSE32646[https://www.ncbi.nlm.nih.gov/geo/query/acc.cgi?acc=GSE32646]). The RNAseq and mutation data for the CALGB40601 clinical trial dataset are retrieved from dbGaP (phs001570.v2.p1[https://www.ncbi.nlm.nih.gov/projects/gap/cgi-bin/study.cgi?study_id=phs001570.v2.p1]) that are available under restricted access controlled by the NCI Data Access Committee [NCIDAC@mail.nih.gov]. A minimum dataset compendium containing the TCGA, GDSC, CCLE, BATTLE, and French CIT datasets is made available through Zenodo[https://zenodo.org/badge/latestdoi/444456261]

Field-specific reporting

Please select the one below that is the best fit for your research. If you are not sure, read the appropriate sections before making your selection.

☒ Life sciences ☐ Behavioural & social sciences ☐ Ecological, evolutionary & environmental sciences

For a reference copy of the document with all sections, see nature.com/documents/nr-reporting-summary-flat.pdf

Life sciences study design

All studies must disclose on these points even when the disclosure is negative.

Sample size	The sample sizes of the analyses are determined based on available data provided by published datasets
Data exclusions	The treatment arm with less than 10 subjects are excluded from our analysis. To reduce the complexity of the manuscript, some minor data not related to the main conclusion of the present study are excluded.
Replication	not applicable.
Randomization	The training and testing sets used for iGenSig modeling are generated from the GDSC profiled cell lines based on 5 randomizations.
Blinding	All datasets are from public repositories thus this study is not blinded.

Reporting for specific materials, systems and methods

We require information from authors about some types of materials, experimental systems and methods used in many studies. Here, indicate whether each material, system or method listed is relevant to your study. If you are not sure if a list item applies to your research, read the appropriate section before selecting a response.

Materials & experimental systems

n/a	Involved in the study	
☒	☐	Antibodies
☒	☐	Eukaryotic cell lines
☒	☐	Palaeontology and archaeology
☒	☐	Animals and other organisms
☒	☐	Human research participants
☒	☐	Clinical data
☒	☐	Dual use research of concern

Methods

n/a	Involved in the study	
☒	☐	ChIP-seq
☒	☐	Flow cytometry
☒	☐	MRI-based neuroimaging
